# Intraintestinal fermentation of fructo- and galacto-oligosaccharides and the fate of short-chain fatty acids in humans

**DOI:** 10.1016/j.isci.2024.109208

**Published:** 2024-02-10

**Authors:** Mara P.H. van Trijp, Melany Rios-Morales, Ben Witteman, Fentaw Abegaz, Albert Gerding, Ran An, Martijn Koehorst, Bernard Evers, Katja C.V. van Dongen, Erwin G. Zoetendal, Henk Schols, Lydia A. Afman, Dirk-Jan Reijngoud, Barbara M. Bakker, Guido J. Hooiveld

**Affiliations:** 1 Division of Human Nutrition and Health, Wageningen University, Wageningen 6708 WE, the Netherlands; 2 Laboratory of Pediatrics, Center for Liver, Digestive and Metabolic Diseases, University of Groningen, University Medical Center Groningen, Groningen 9713 GZ, the Netherlands; 3 Hospital Gelderse Vallei, Department of Gastroenterology and Hepatology, Ede 6716 RP, the Netherlands; 4 Statistics and Probability Unit, University of Groningen, Groningen 9747 AG, the Netherlands; 5 Department of Laboratory Medicine, University of Groningen, University Medical Center Groningen, Groningen 9713 GZ, the Netherlands; 6 Laboratory of Microbiology, Wageningen University, Wageningen 6708 WE, the Netherlands; 7 Division of Toxicology, Wageningen University, Wageningen 6708 WE, the Netherlands; 8 Laboratory of Food Chemistry, Wageningen University, Wageningen 6708 WG, the Netherlands

**Keywords:** Human metabolism

## Abstract

Consumption of fructo- (FOS) and galacto-oligosaccharides (GOS) has health benefits which have been linked in part to short-chain fatty acids (SCFA) production by the gut microbiota. However, detailed knowledge of this process in the human intestine is lacking. We aimed to determine the acute fermentation kinetics of a FOS:GOS mixture in healthy males using a naso-intestinal catheter for sampling directly in the ileum or colon. We studied the fate of SCFA as substrates for glucose and lipid metabolism by the host after infusion of ^13^C-SCFA. In the human distal ileum, no fermentation of FOS:GOS, nor SCFA production, or bacterial cross-feeding was observed. The relative composition of intestinal microbiota changed rapidly during the test day, which demonstrates the relevance of postprandial intestinal sampling to track acute responses of the microbial community toward interventions. SCFA were vividly taken up and metabolized by the host as shown by incorporation of ^13^C in various host metabolites.

## Highlights

• Fructo- and galacto-oligosaccharide fermentation inside the human intestine was studied
• No fermentation or SCFA production was observed in the distal small intestine
• The small intestine microbiota changed rapidly after consumption of the oligosaccharides
• Intestinal-delivered ^13^C-SCFAs were vividly taken up and metabolized by the host

## Graphical abstract

**Figure:**
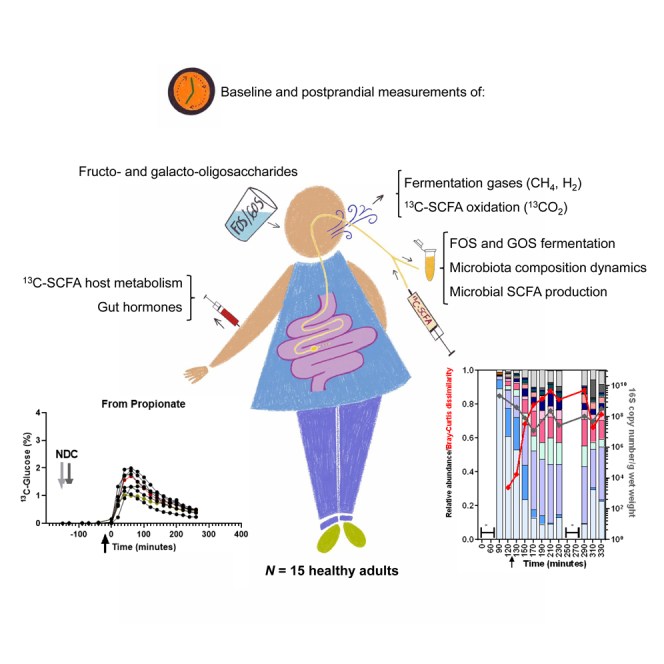

## Introduction

The incidence of obesity and diabetes has rapidly increased, making them worldwide public health problems.^1^ Consumption of non-digestible carbohydrates (NDC, 25–50 g/day) is associated with numerous health benefits, including decreased risk of diabetes and obesity.^2^ Many NDC can be fermented by the intestinal microbiota, and some NDC, such as fructo- and galacto-oligosaccharides (FOS and GOS, respectively) have been shown to selectively increase specific groups of bacteria such as *Bifidobacterium*.^3^ During NDC fermentation, short chain fatty acids (SCFA) are produced, mainly acetate, propionate, and butyrate.^4^ Furthermore, metabolic cross-feeding by bacteria plays a key role in maintaining the microbial ecosystem and is crucial to determine the final SCFA profile in the intestine.^5^ It has been hypothesized that SCFA are an important link between NDC fermentation and host health improvements.^6^^,^^7^ To avoid invasive sampling, feces are mostly used when studying NDC degradation, SCFA production, and intestinal microbiota in humans. However, feces are expected to only be a surrogate representation of the microbiota residing in the lumen.^8^^,^^9^ Moreover, colonic SCFA are readily absorbed, with likely only 5–10% being excreted in the feces.^10^ Additionally, NDC degradation and SCFA production is often studied in *in vitro* gut models that can reproduce physicochemical parameters of the human gut, however, SCFA absorption in this model does not directly mimic luminal dynamics.^11^^,^^12^ Furthermore, *in vitro* models are mostly inoculated with human feces, results of which cannot be directly translated to the *in vivo* situation in humans. Another indirect method to measure carbohydrate fermentation in healthy subjects is by isotopic labeling of the substrate of interest, such as ^13^C-inulin,^13^ followed by tracking postprandial ^13^C-metabolites in plasma as an indication of carbohydrate degradation, or the use of hydrogen and methane concentrations in the breath as an indicator of microbial fermentation.^14^^,^^15^^,^^16^^,^^17^ Still, this does not provide us with *direct* information about carbohydrate fermentation and SCFA production inside the human intestinal lumen, nor on the direct impact of NDC on the luminal microbiota.

In mice fed with diets supplemented with different amounts of fermentable fiber (guar gum), only the *in vivo* uptake fluxes of SCFA, and not their cecal concentrations, correlated linearly with the improvements in metabolic syndrome markers.^18^ To better understand the health benefits of fiber fermentation, accurate estimation of SCFA production, uptake and their metabolic fate in the host is essential. Assessing such kinetics of fermentation *in vivo* in humans comes with major challenges. Boets et al. pioneered such studies, by measuring SCFA appearance in blood derived from inulin ingestion after a continuous intravenous infusion of ^13^C-SCFA,^19^ or by directly delivering the labeled SCFA in the proximal colon using capsules with a pH-responsive coating and measuring the appearance of label in the blood.^20^ However, no samples were obtained from the actual luminal fermentation site. The measurement of feces, blood, and breath necessitates assumptions for the calculation of intestinal fluxes that are debatable, since there might be a significant first-passage clearance of luminal SCFA before reaching peripheral blood circulation,^21^ which may blunt luminal SCFA production and interconversion estimates. Moreover, SCFA can have a local effect for instance working as signaling molecules to promote the release of satiety-inducing hormones such as glucagon-like peptide 1 (GLP-1) and peptide YY (PYY).^22^ They are also metabolized locally by colonocytes in the gut, or after absorption into the portal circulation by the liver.^23^ In humans and mice, SCFA can be used as precursors for glucose and lipids by the host.^20^^,^^24^ Nonetheless, the mechanisms by which SCFAs can regulate host metabolism and to which extent they could be beneficial in humans need to be further studied. Here, we present the results of two clinical feasibility trials in healthy men. The first trial aimed to study acute NDC fermentation kinetics in the (small) intestine in humans using a novel approach based on intestinal catheters for *in situ* sampling and administration of ^13^C labeled SCFA. The same methodology was implemented in the second trial, where we aimed to investigate the effect of a 7-day NDC supplementation versus maltodextrin on the acute NDC fermentation kinetics. The use of stable isotopes allowed us to assess the fate of intestinal-delivered SCFA as substrates in systemic glucose and lipid metabolism. Our studies provide valuable information about the feasibility of this new approach to study bacterial NDC fermentation inside the human intestine and the fate of fermentation products.

## Results

### Study logistics and subject characteristics

Two out of five subjects fully completed study 1 (no intervention), and six out of ten subjects (n = 4 NDC group, n = 2 placebo group) fully completed study 2 (Figure S2). Five subjects dropped out due to failure of proper catheter placement in the distal ileum or colon and two subjects dropped out due to adverse events during the study. More details on the individual studies, including study flow charts and drop-outs, can be found in the Supplementary Results. The baseline characteristics between studies and groups were similar (Table 1).

**Table 1:** Baseline characteristics and habitual daily intake of (macro)nutrients in healthy male subjects

	Study 1: no intervention (n = 2)	Study 2: NDC group (n = 4)	Study 2: placebo group (n = 2)	p-value
Age, y^a^	39.5 ± 18.5	26.0 ± 8.2	39.0 ± 28.3	0.939
BMI, kg/m^2^	25.3 ± 0.8	22.0 ± 0.7	24.2 ± 5.4	0.363
Total kcal/day	2648.6 ± 79.1	2436.1 ± 235.3	3092.1 ± 616.5	0.205
Total carbohydrates, g/day^b^	294.1 ± 35.4	239.8 ± 31.2	285.2 ± 50.0	0.205
Mono- and disaccharides, g/day	94.9 ± 19.1	83.7 ± 48.5	112.9 ± 25.9	0.472
Polysaccharides, g/day	199.2 ± 16.3	155.9 ± 17.5	172.2 ± 24.1	0.210
Fiber, g/day^c^	36.5 ± 9.6	23.9 ± 2.2	32.4 ± 0.9	0.069
Total protein, g/day	101.9 ± 0.3	92.8 ± 19.1	112.7 ± 13.3	0.248
Total fat, g/day	99.2 ± 11.8	104.5 ± 20.4	141.0 ± 41.1	0.248
Alcohol, g/day	12.2 ± 2.1	15.8 ± 4.3	21.7 ± 0.7	0.097

a Values are presented as group means ± SD.

b Total carbohydrates does not include dietary fiber.

c Included are high molecular weight fibers (e.g., cellulose, resistant starch, cereal β-glucan, guar gum, and certain xylans), insoluble fibers in water (e.g., cellulose, resistant starch, and certain xylans), fibers soluble in water and precipitated by 78% ethanol (e.g., cereal β-glucan, guar gum, and certain xylans). Excluded are low molecular weight fibers (e.g., fructan, GOS, polydextrose, and resistant maltodextrins), and non-resistant starch.

**Figure 1:**
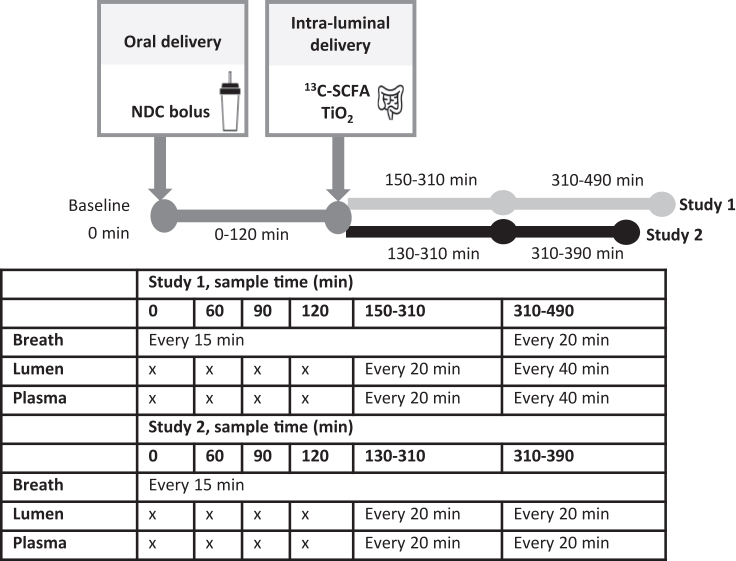
Schematic overview of the experimental test day Breath samples, intestinal luminal content, and blood were collected over time. Differences in the timeline between study 1 and study 2 are indicated in light gray and black, respectively. Abbreviations: FOS, fructo-oligosaccharides; GOS, galacto-oligosaccharides; NDC, non-digestible carbohydrates; TiO_2_, titanium dioxide; SCFA, short-chain fatty acids.

Although we aimed to sample from a uniform location in all subjects, namely the proximal colon as main NDC fermentation site, in practice this was not feasible due to placement difficulties and clogging of tube when sampling. The intestinal regions that were studied included the distal ileum (n = 6), proximal colon (n = 1), and transverse colon (n = 1) (Table 2). Luminal sampling was only possible after NDC consumption, but was in some individuals still not possible at every envisioned time point, see Figure 1.

**Table 2:** Intestinal regions studied in the participants

Subject	Study and intervention	Catheter distance from the nose	Catheter tip location	Estimated distance from the ileo-cecal valve^a^	Number of collected aspirates	Sampling duration (minutes)^b^
S1	1, None	240 cm	Distal ileum	15 cm	14	4.4 (3.6)
S2	1, None	230 cm	Distal ileum	25 cm	6	15.0 (6.0)
S3	2, Placebo	270 cm	Proximal colon	10 cm	5	8.0 (5.5)
S4	2, Placebo	300 cm	Transverse/descending colon	–	4^#^	5.0 (1.3)^#^
S5	2, NDC	270 cm	Distal ileum	<25 cm	9	3.0 (0.9)
S6	2, NDC	290 cm	Distal ileum	10 cm	5	5.0 (6.0)
S7	2, NDC	240 cm	Distal ileum	<50 cm	13	2.0 (3.5)
S8	2, NDC	275 cm	Distal ileum	10 cm	13	5.0 (2.0)

a As calculated from the fluoroscopy pictures that were taken after the delivery of contrast liquid inside the intestine.

b the time required to take a 2–2.5 mL sample, in minutes as median and inter-quartile range ^#^samples could only be collected after infusion of saline solution in the aspiration channel to dilute the aspirate.

**Figure 2:**
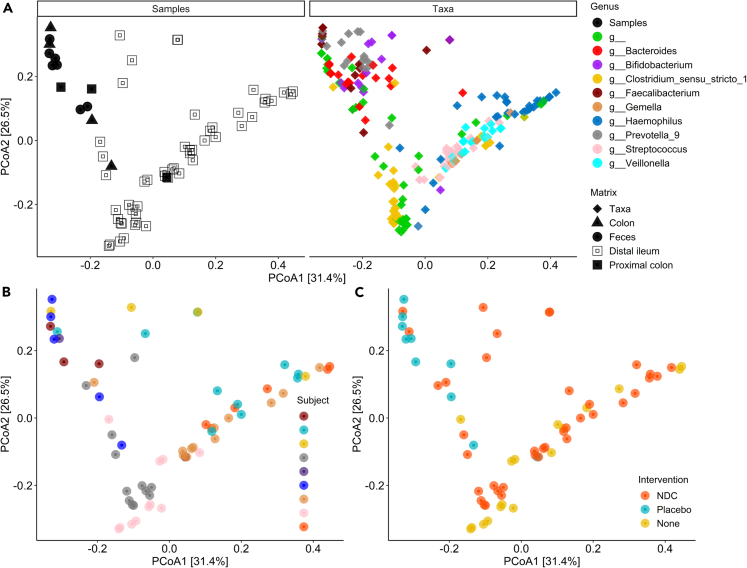
The overall microbiota variation between the samples in the dataset Principle coordinate analysis plots based on weighted UniFrac (beta-diversity) of (A) samples from the different matrixes (distal ileum, proximal colon, transverse colon, and feces) in the left facet, and in the right facet the contributions of the top 10 bacteria genera shown on the same graphical space, (B) visualization of the microbiota variation between subjects, (C) visualization of the microbiota variation between the intervention groups. All luminal samples are shown, including multiple time points per subject. Data are presented from n = 9 subjects in study 1 and study 2.

As shown in Table 2, all measurements in the distal ileum were performed in subjects in study 1 without intervention (n = 2) or in those who consumed NDC supplements for 7 days (n = 4) in study 2, while the measurements in the colon were performed in subjects that consumed placebo supplements for 7 days (n = 2) in study 2. The microbiota composition was distinct between sampling sites (i.e., distal ileum versus colon and feces), while no clusters, i.e., a more comparable intestinal microbiota, were revealed based on the dietary intervention groups (Figure 2). In the ileum, the bacteria *Haemophilus, Clostridium sensu stricto 1, Streptococcus, Enterobacteriaceae_g_*_, and *Veillonella* were more abundant than in the colon and feces, while in the colon and feces the relative abundances of *Bifidobacterium, Dialister, Prevotella_9, Bacteroides, and Lachnospiraceae_g__* were higher (Figure 2A; Table S1). Also, the fecal microbiota and SCFA were not different between intervention groups (Table S2). Since the main outcome, acute NDC fermentation *in vivo*, strongly depends on the luminal microbiota composition at the sampling location, we decided to combine the data per sampling site.

### Intra-intestinal non-digestible carbohydrates fermentation over time

**Figure 3:**
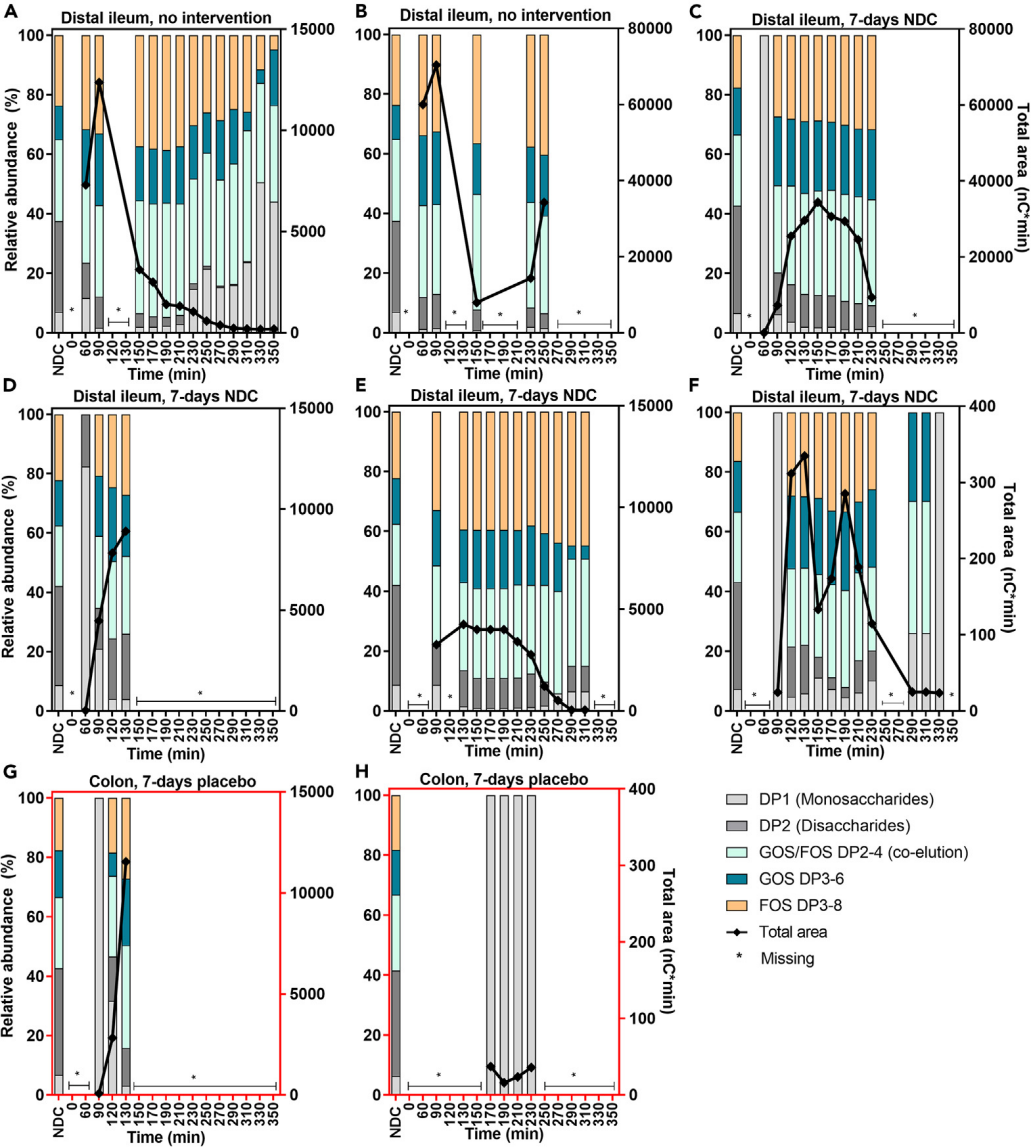
Non-digestible carbohydrates in the distal ileum or colon of healthy males over time The relative abundances of each constituent of the NDC bolus are shown on the left y axis. The black line represents the total area of all NDC constituents, indicated on the right y axis. ∗ For this time point, a sample could not be obtained due to sampling difficulties. DP, degree of polymerization; FOS, fructo-oligosaccharides; GOS, galacto-oligosaccharides. Data are presented from the n = 8 subjects in study 1 and study 2.

To assess acute fermentation kinetics in the intestine, we investigated intestinal NDC degradation and NDC-induced SCFA production and interconversions over time. The constituents present in the NDC bolus (DP1-DP8) were detected in the distal ileum of all subjects starting 60–120 min after consumption (Figure 3). No changes in ratios of NDC DP ≥ 3 were found in the distal ileum over time when compared to those ingested via the NDC bolus. Only after 210–250 min, monosaccharides (DP1) were increased relative to first time points in the ileum of two subjects (Figures 3A and 3F), but the total amount of the NDC was very low at these time points (Figure 3, black lines). The changes over time of total NDC peak area in the intestine followed that of the non-absorbable marker PEG-4000 (Figure S3), suggesting removal from the sampling site via intestinal peristalsis rather than fermentation.

**Figure 4:**
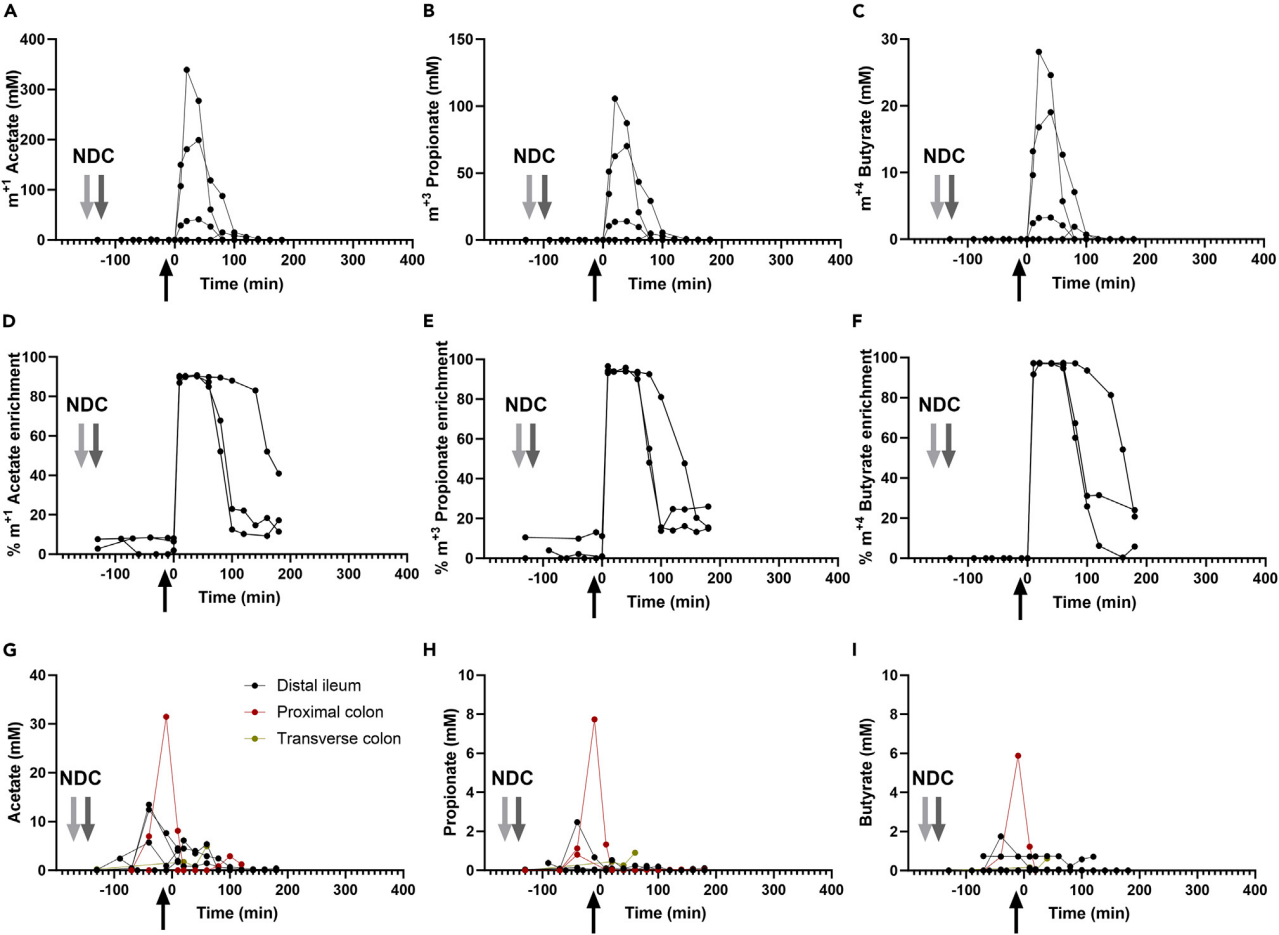
Luminal SCFA enrichments and concentrations in the intestinal samples of healthy male subjects ^13^C- SCFA concentrations (A–C) and enrichments (D–F) over time from the 3 subjects from whom luminal samples could be collected before and after isotope delivery (all these 3 subjects had the catheter tip located in the distal ileum). Non-labeled SCFA concentrations (G–I) from all subjects over time. The lines represent the individual subjects separately. The black arrow indicates the start of luminal isotope infusion (10 mL containing 10 mmol [1-^13^C_1_]-acetate, 4 mmol [1,2,3-^13^C_3_]-propionate and 1 mmol [1,2,3,4-^13^C_4_]-butyrate) through the catheter. This is considered time 0 for all subjects to match both studies. The gray arrows indicate the drinking of the non-digestible carbohydrates (NDC) bolus in both studies (−150 min for study, light gray arrow; and −120 min for study 2, dark gray arrow). Data are presented from n = 4 subjects in study 1 and study 2 for which sufficient material for analyses could be obtained.

Luminal SCFA concentrations and ^13^C-SCFA enrichment were measured to determine bacterial SCFA production and interconversion induced by NDC fermentation (Figure 4). It is important to note that we only present the luminal isotope data (Figures 4A–4F) where samples after isotope delivery could be obtained. This included 3 subjects where samples were collected in the distal ileum. First, we examined SCFA interconversion by measuring all different label patterns of acetate, propionate, and butyrate. Besides the isotopologues to be expected from the delivered SCFA (acetate m^+1^, propionate m^+3^, and butyrate m^+4^) no other label patterns were found, suggesting no interconversion of SCFA by ileum microbiota (Figures 4A–4F). The delivery of labeled SCFA resulted in a steep increase and subsequent decrease of the luminal concentration of ^13^C-SCFA, indicating its appearance by infusion and its disappearance from the sampling location in the distal ileum (Figures 4A–4C). The luminal kinetics of all three SCFA were similar, and their mass ratios in the luminal samples remained similar to those delivered (Figures 4A–4C). The increase in ^13^C-SCFA concentration was accompanied by an almost instantaneous increase of ^13^C-SCFA enrichment of the delivered isotopologues (i.e., ^13^C relative to total) reaching a plateau at nearly 100% of ^13^C-C enrichment (Figures 4D–4F). Moreover, the ^13^C enrichment only decreased when its concentration approached the pre-delivery level (Figures 4A–4C) and there was no significant increase in SCFA from NDC fermentation (Figures 4G–4I). This was in line with the lack of NDC degradation in the distal ileum. During the test day, from two subjects samples were obtained from the proximal colon and the distal colon, but only before ^13^C-SCFA delivery. In the proximal colon, non-labeled SCFA concentrations increased up to ∼30 mM acetate, 8 mM propionate, and 6 mM butyrate after 120 min of NDC bolus consumption (Figures 4G–4I). In the transverse colon, SCFA concentrations did not increase, likely due to dilution of the sample after saline delivery through the aspiration channel to decrease sampling clogging. Overall, NDC degradation and SCFA production by the distal ileum bacteria was minimal and there was no bacterial SCFA cross-feeding during the time the isotopes remained in the sampling location.

### Microbial dynamics in the intestine over time

**Figure 5:**
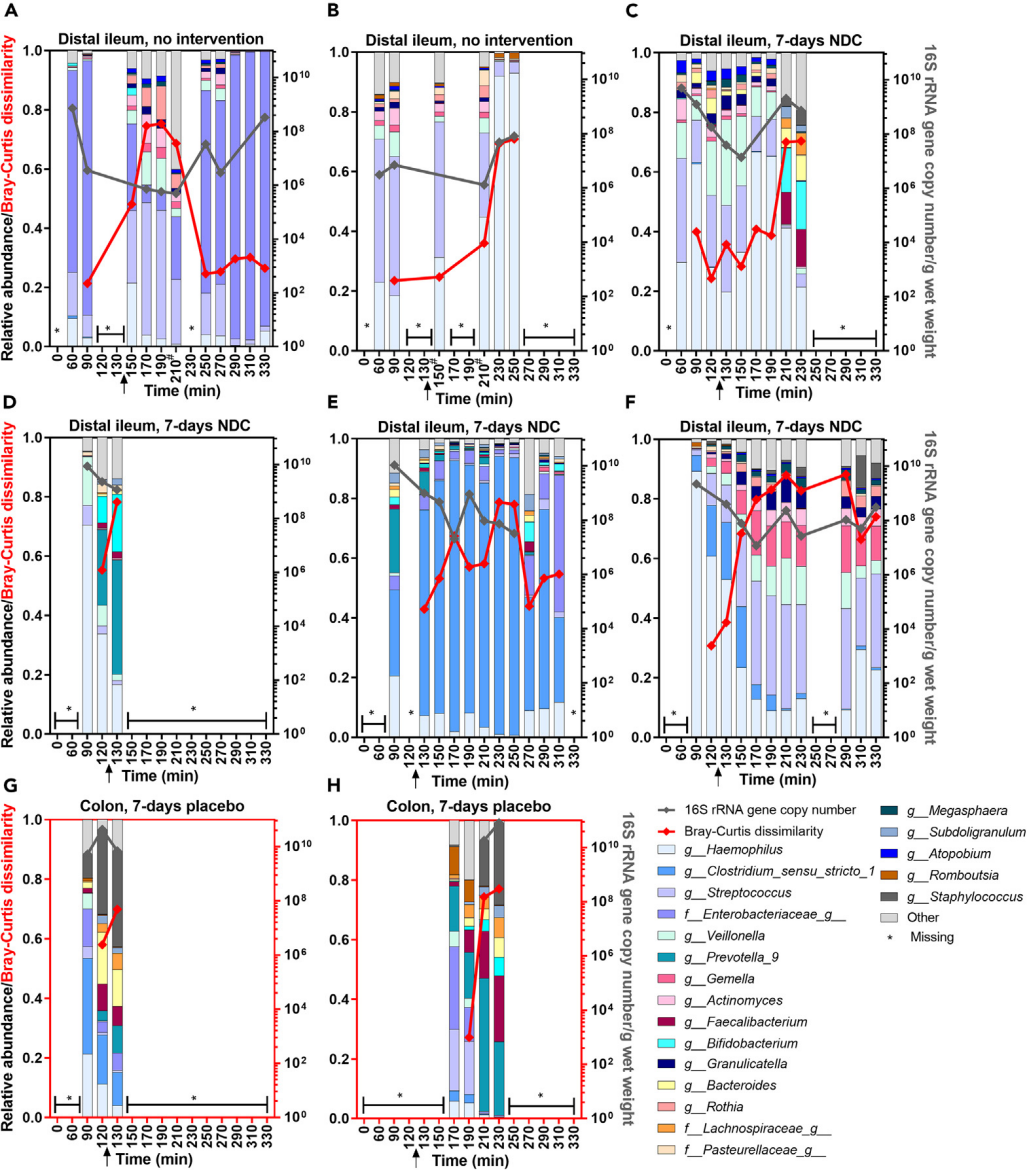
The microbiota, the microbiota dissimilarity, and total 16S rRNA gene copy number in distal ileum or colon of healthy male subjects over time after consumption of the NDC bolus The top 20 bacteria on genus level are shown. The arrow shows the moment of intra-intestinal infusion (^13^C-SCFA/TiO_2_) close to the sampling location. The right y axis indicates the total 16S rRNA gene copy number which is depicted by the gray line (missing values were due to low availability of DNA). The Bray-Curtis dissimilarity values are visualized by the red line on the left y axis, which show the dissimilarity of the microbiota at each time point compared to the microbiota in the first collected sample. Bray–Curtis dissimilarity values range between 0 and 1, when closer to 1 means the two samples do not share any bacteria. ∗For this time point, a sample could not be obtained due to sampling difficulties. ^#^Samples contained less than 10,000 sequencing reads. Data are presented from the n = 8 subjects in study 1 and study 2.

Although no fermentation at the small intestinal sampling location was detected by our experimental setup, we studied whether the consumption of NDCs affected the intestinal microbiota during the test day. All samples passed the quality control for microbiota analyses (Figure S4). On the individual level, the relative microbiota composition in the intestinal samples changed rapidly over time, as indicated by the Bray-Curtis dissimilarity values that increased during the day when compared to the first collected sample (Figure 5, red line). Even though the relative microbiota composition changed, the total bacteria numbers, signified by analyses of 16S rRNA gene copy numbers, did not increase over time. Moreover, selected and detected bacteria known to be stimulated by the provided FOS or GOS, namely *Bifidobacterium*, *Lactobacillus*, *Streptococcus*, and *Bacteroides*, did not significantly increase over time (Figure S5).

To evaluate whether the lack of fermentation in the distal ileum was the result of the lack of fermentation capacity of the ileum microbiota, we compared the microbial pathways related to fermentation between the ileum and the feces (Table 3), since it has been shown previously that fecal microbiota more efficiently ferments FOS and GOS *in vitro*. The specific microbial pathways were derived from the total predicted genomes, predicted based on the 16S rRNA gene sequencing outcomes. Indeed, three microbial fermentation pathways were about 2-fold lower in relative abundance in the ileum compared to the colon and/or feces (p < 0.05).

**Table 3:** The relative abundances (%) of selected microbial fermentation pathways in the predicted microbial genome of luminal content or feces of healthy male subjects

Microbial pathway	Pathway number	Relative abundance distal ileum (n = 6 subjects, 52 samples)^a^	Relative abundance colonic content (n = 2 subjects, 7 samples)	Relative abundance feces (n = 7 subjects)	p-value^b^
*Pyruvate fermentation to acetate and lactate II*	PWY-5100	0.790 ± 0.302	0.761 ± 0.082	0.771 ± 0.035	0.857
*Pyruvate fermentation to propanoate I/succinate-propionate fermentation pathway*	P108-PWY	0.134 ± 0.102	0.269 ± 0.089	0.248 ± 0.132	0.002^c^
*Acetyl-CoA fermentation to butanoate II/butyrate II*	PWY-5676	0.082 ± 0.063	0.175 ± 0.060	0.164 ± 0.087	0.000^c^
*Pyruvate fermentation to butanoate/butyrate*	CENTFERM-PWY	0.071 ± 0.063	0.153 ± 0.060	0.081 ± 0.057	0.014^d^
*Succinate fermentation to butanoate/butyrate*	PWY-5677	0.015 ± 0.015	0.010 ± 0.009	0.009 ± 0.008	0.690
*Bifidobacterium shunt/glucose fermentation to lactate*	P124-PWY	0.111 ± 0.084	0.082 ± 0.033	0.243 ± 0.176	0.086

a Data are presented as mean relative abundance (%) ± SD.

b The relative abundances in the ileum were compared to those in the feces or colon using an independent samples Kruskal-Wallis test. Because of the high variability in microbiota composition during the day, all samples collected *in vivo* are treated as independent observation.

c Ileum samples were significantly different from both colon and feces samples.

d ileum samples were significantly different from colon samples.

### Systemic biomarkers for fiber fermentation: Breath gases and blood SCFA

**Figure 6:**
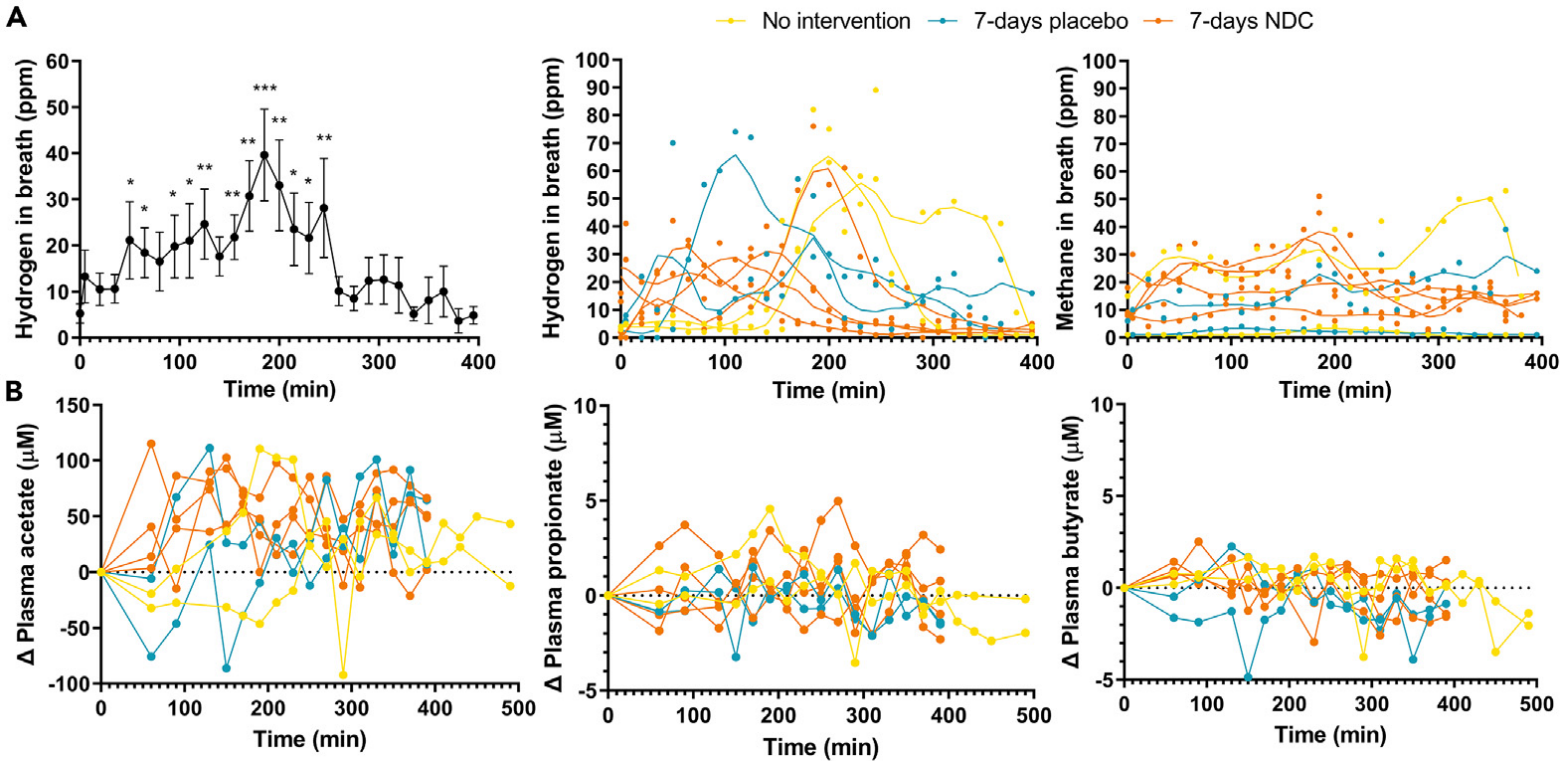
Biomarkers of NDC fermentation (A) Breath concentrations of methane and hydrogen and (B) the changes of SCFA in the blood of healthy male subjects after consumption of the NDC bolus with 10 g FOS and GOS. Data are represented as mean ± SD, n = 8 subjects in study 1 and study 2, or as the individual responses. LOWESS (Locally Weighted Scatterplot Smoothing) curves plot was applied to the breath hydrogen and methane to show the general trends over time, with 10 data points in the smoothing window. Significance is shown as ∗ = p < 0.05, ∗∗ = p < 0.01, ∗∗∗ = p < 0.001 compared to baseline. NDC, non-digestible carbohydrates. Data are presented as mean ± SD, n = 8 subjects in study 1 and study 2, or as the individual responses of the n = 8 individuals.

To determine if fermentation occurred beyond the sampling site, systemically available markers for NDC fermentation, namely H_2_ and CH_4_ in breath, and SCFA in blood were evaluated after NDC consumption. Breath hydrogen was significantly increased between 50 and 245 min after consumption of FOS and GOS compared to baseline (Figure 6A), with a peak mean concentration (±SD) of 39.6 ± 28.1 ppm at 185 min. The start of fermentation, as indicated by increased breath hydrogen, was highly variable between subjects (35–170 min), while breath CH_4_ did not significantly increase over time after NDC consumption. The measurement of SCFA in the blood (Figure 6B) over time showed that acetate increased over time (p = 0.003, baseline: 56 ± 39 μM, 330 min: 120 ± 51 μM), while propionate and butyrate did not significantly change compared to baseline (baseline: 1.96 ± 1.28 μM and 5.03 ± 1.20 μM, respectively). Since we did not measure fermentation in the distal ileum *in vivo*, these increased markers point to fermentation of FOS and GOS beyond the sampling site, likely in the colon.

### The role of SCFA in host metabolism

**Figure 7:**
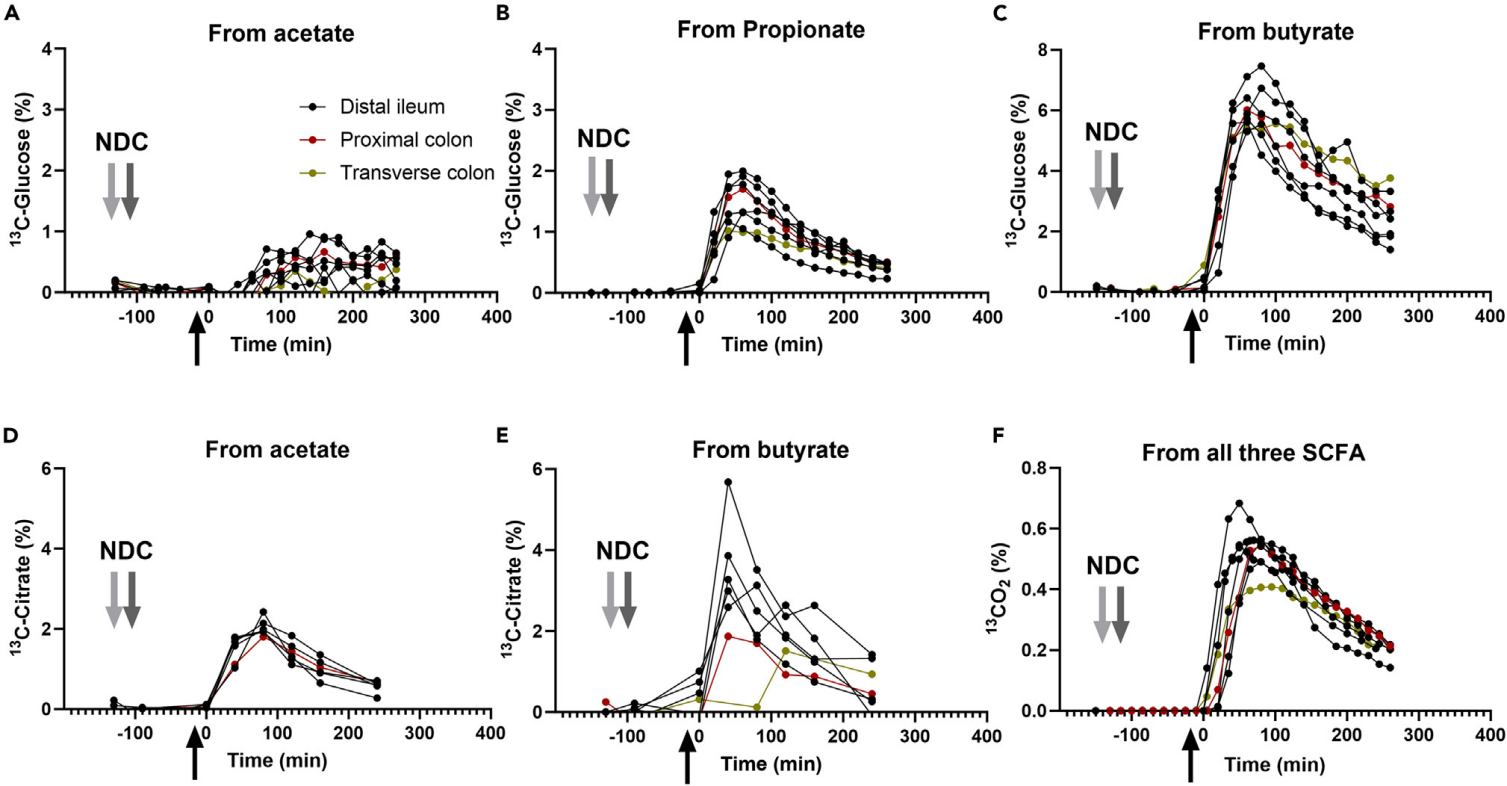
Assimilation of ^13^C-SCFA into glucose and acyl-carnitines in blood and its oxidation to ^13^CO_2_ in the breath (A–C) Glucose and (D–E) citrate enrichment from labeled acetate, propionate, or butyrate, delivered in the distal ileum, proximal colon, or distal colon. (F) ^13^C incorporation into CO_2_ over time from all three ^13^C-SCFA together delivered in the distal ileum, proximal colon, or distal colon. The black arrow indicates luminal isotope infusion through the catheter. This is considered to be time 0 for all subjects to match both studies. The gray arrows indicate the consumption of the non-digestible carbohydrates (NDC) bolus in both studies (−150 min for study 1, light gray arrow, and −120 min for study 2, dark gray arrow). Data are presented from n = 8 subjects in study 1 and 2, of which n = 6 subjects for distal ileum, n = 1 for proximal colon, and n = 1 for the distal colon.

SCFA can function locally at their production site as signaling molecules by promoting the release of satiety hormones. Before and over time after consumption of the NDC bolus, the total PYY concentrations (p = 0.92) and the total GLP-1 (p = 0.37) concentrations did not change over time (Figure S6). To study SCFA metabolization in the human body, we measured the label incorporation from the delivered ^13^C-SCFA into plasma glucose, organic acids, amino acids, acyl-carnitines, fatty acids, and into breath CO_2_. Label incorporation in plasma glucose rapidly appeared after isotope infusion with M^+2^ > M^+1^ > M^+3^ (Figure S7A). The different isotopologue distributions were first calculated back to the SCFA source and normalized to the delivered amount (Supplementary Methods). All three SCFA transferred ^13^C to glucose, with butyrate being the most efficient (Figures 7A–7C). Glucose concentrations remained constant over time for most of the subjects (Figure S7B). The organic acid measurement showed an increase in citrate enrichment, mostly as M^+1^ and M^+2^ coming from acetate and butyrate, respectively (Figure S7C). After normalizing by source and delivered amounts, butyrate was found to be also the most efficient contributor to citrate enrichment (Figures 7D and 7E). We did not find any enrichment in plasma amino acids and fatty acids. As a proxy of fatty-acid metabolism, the different enrichments patterns in the different acyl-carnitines were measured in plasma. The only enrichment found was M^+3^ in propionyl-carnitine (Figure S7D). The most likely source of M^+3^ propionyl-carnitine enrichment is [1,2,3-^13^C_3_]-propionate that can be bound to carnitine and released into the circulation. To estimate whole-body SCFA metabolism, ^13^C label incorporation into CO_2_ generated from ^13^C-substrates was measured in exhaled breath after isotope infusion. CO_2_ was immediately incorporated the label and ^13^CO_2_ slowly decreased over time (Figure 7F). This ^13^CO_2_ enrichment curve initially represents the direct oxidation of all three luminal SCFA (since the specific source SCFA cannot be identified in this case) and at the later time points the oxidation of the secondary metabolites derived from the luminal SCFA as substrates, such as glucose. Together, these observations show that delivered SCFA were extensively taken up and metabolized by the host.

### Kinetics of ^13^C-SCFA incorporation into glucose

**Figure 8:**
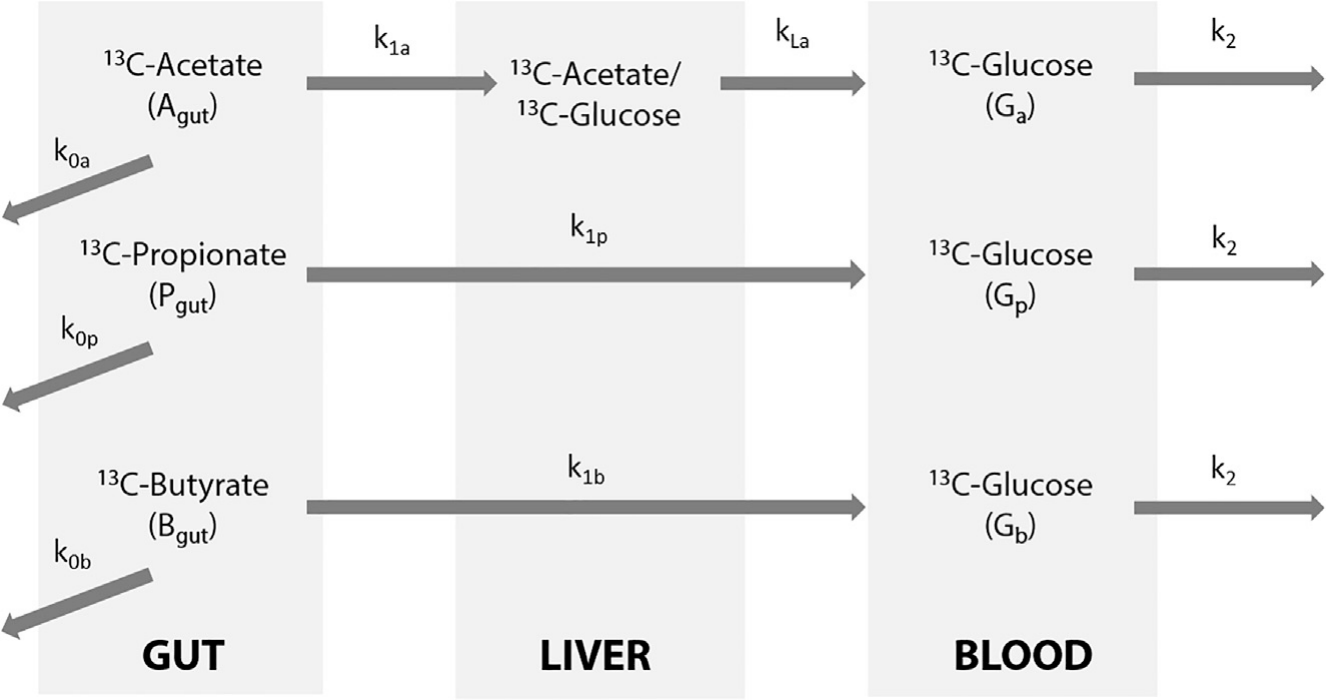
Schematic overview of the three-compartment model of the incorporation of ^13^C-SCFA into glucose

To describe the kinetics of incorporation of each individual SCFA into glucose, a three-compartment model consisting of the gut, the liver, and the blood plasma was constructed (Figure 8). The model describes.

(i) Uptake of each ^13^C-SCFA from the gut, characterized by apparent rate constants k_1a_, k_1p_, and k_1b_ for acetate, propionate, and butyrate respectively;
(ii) Incorporation of ^13^C into glucose, which is lumped with uptake in the case of propionate and butyrate (k_1p_ and k_1b_), but characterized by a distinct rate constant k_LA_ in the case of acetate;
(iii) Loss of each of the ^13^C-SCFA to other fates than incorporation into glucose, due to for example excretion, CO_2_ production, or conversion into other metabolites, characterized by rate constants k_0a,_ k_0p_ and k_0b_; and
(iv) Clearance of ^13^C-glucose from the plasma compartment with a single rate constant k_2_*,* irrespective of the source of the ^13^C in the glucose.

The additional liver compartment was only included for acetate, since the kinetics of incorporation of ^13^C from acetate into glucose were more complex than that of the other SCFA (Figures 7A–7C) and could not be fitted otherwise.

**Figure 9:**
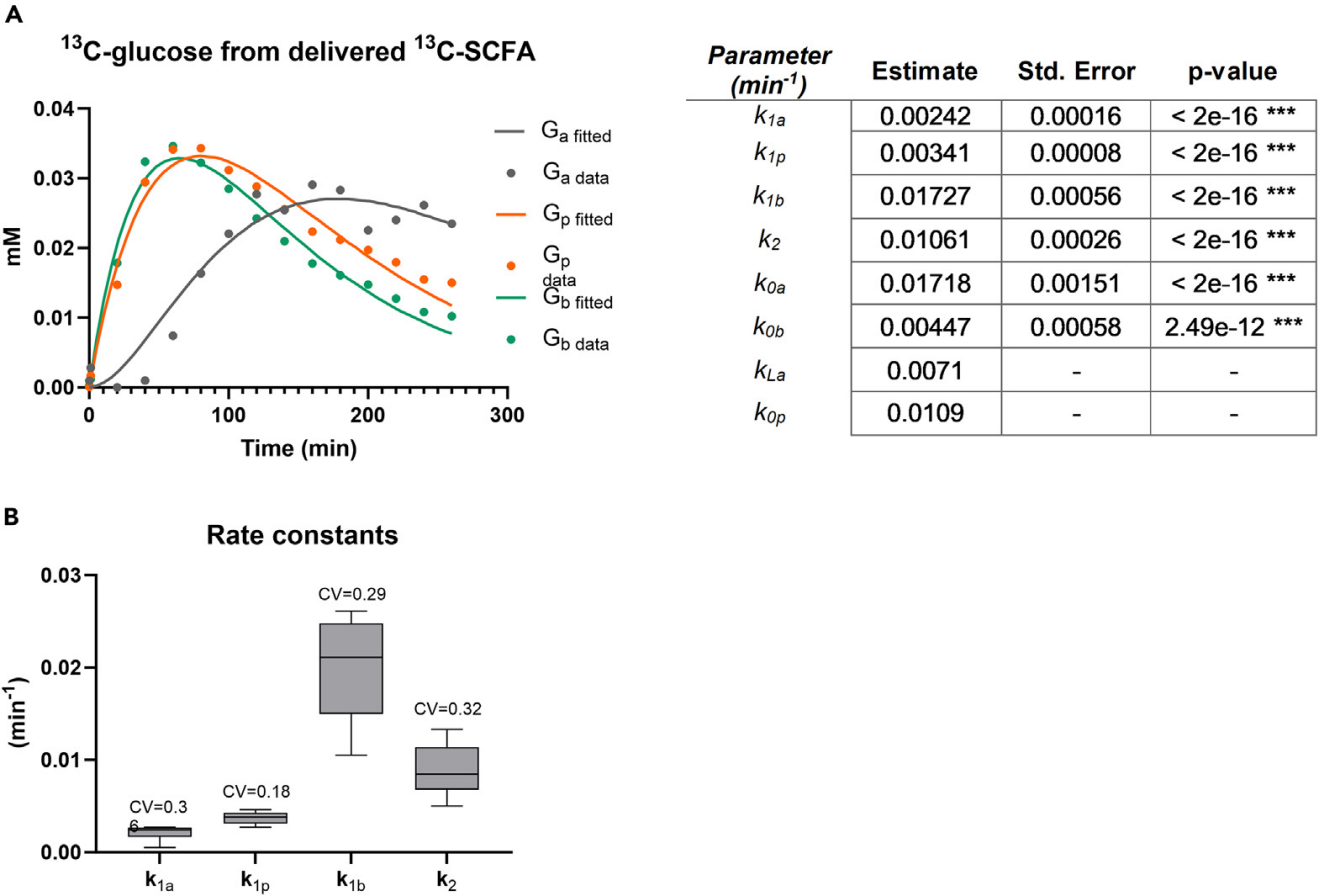
Model fitting for the average data of all subjects (A) Experimentally measured values (in dots) and fitted values (in lines) of the incorporation of ^13^C-labeled carbon atoms of delivered labeled acetate, propionate and butyrate into blood glucose over time and the estimated constant rates. (B) K_La_ and k_0p_ were fixed at 0.0071 and 0.0109 min^−1^, respectively. ∗ = p < 0.05. ∗∗ = p < 0.01. ∗∗∗ = p < 0.001. Data are presented from n = 8 subjects in study 1 and study 2.

The model was translated into a set of ordinary differential equations. Assuming a constant plasma volume of 0.15 L per kg body weight,^45^ six out of the eight rate constants could be reliably fitted from the time courses of labeled glucose (Figure S8), for either the average of all subjects (Figure 9A) or each subject separately (Figures S9–S16). Significant identification of the rate constants is indicated by the small p values (Figure 9A). The rate constant of butyrate incorporation into glucose (k_1b_) was found to be the highest, while its specific loss (k_0b_) was the lowest. Based on the coefficients of variation the uptake rate constant of acetate (k_1a_) was most dispersed, while that of propionate (k_1p_) was most similar between subjects (Figure 9B). The fractions of the delivered ^13^C-acetate, ^13^C-propionate, and ^13^C-butyrate that were incorporated into glucose, were calculated as the ratio between the conversion rates to the sum of the total rates. This was in line with the pharmacodynamic formula for fractional dose contribution. Fractional incorporation of labeled carbons of SCFA into glucose was 0.12 for acetate, 0.23 for propionate, and 0.79 for butyrate. This shows that most of the labeled carbon atoms of butyrate were incorporated into glucose, while carbons of acetate appeared only minimally in glucose.

## Discussion

We are the first to apply naso-intestinal catheters to monitor carbohydrate fermentation, SCFA production, and absorption inside the human intestinal lumen, as well as the direct impact of FOS:GOS on the luminal microbiota. Moreover, the fate of SCFA as substrates for host metabolism was assessed using a stable isotope approach. Biomarkers of fermentation, namely breath hydrogen and plasma SCFA, increased upon consumption of FOS:GOS, indicating fermentation, but no changes in luminal NDC breakdown profiles were observed. Moreover, SCFA production was minimal and there was no interconversion of SCFA in the distal ileum. The relative microbiota abundances in the intestine changed dynamically during the test day. SCFA were rapidly metabolized by the host as shown by ^13^CO_2_ enrichment, and incorporation of ^13^C in various host metabolites. Independently of SCFA delivery location through the intestine, assimilation of SCFA was entirely comparable among all subjects, suggesting complete absorption by the host.

### Luminal NDC breakdown, SCFA production and inter-conversion

Chicory FOS and GOS are prebiotics, and both are well fermentable by fecal bacteria *in vitro.*^49^ Moreover, small intestinal bacteria from ileostomy subjects fermented FOS and GOS before 5 h of incubation, namely 29–89% hydrolysis of FOS, 31–82% hydrolysis of GOS [66], and human ileum mucosa bacteria have hydrolytic activities toward FOS and GOS *in vitro* [67]. Yet, we are the first to study the behavior of a mixture of both in humans. The carbohydrate profiles of the higher DP constituents in the distal ileum or proximal colon over time were comparable to those in the NDC bolus. This indicates no or only minor breakdown of NDCs in the ileum, in agreement with two earlier trials using FOS in ileostomy or healthy subjects.^50^^,^^51^ For GOS, no other *in vivo* studies were performed previously. We used an isotope approach to estimate SCFA production. We observed that the enrichment of the ^13^C-SCFA fraction remained close to 100%, which suggested that there was negligible production of unlabeled SCFA from fermentation of FOS and GOS over time inside the distal ileum. This result was in line with the absence of NDC breakdown in the location. While others reported high concentrations of SCFA in ileostomy effluent,^50^^,^^52^ we measured maximally 16 mmol/L SCFAs in the ileum, which was in line with previous data on sudden death human victims that measured only 13 ± 6 mmol/kg SCFAs in the terminal ileum, but 10-fold higher concentrations in the colon.^53^ In both mice^24^ and humans^20^ interconversion of SCFA in the cecum was mainly from acetate to butyrate. In humans, this was previously studied indirectly by measuring labeled-SCFA in blood after intestinal delivery of other individual SCFA.^20^ If SCFA would have been interconverted by bacterial cross-feeding, we should have seen other SCFA isotopologues than we had administered. However, no changes in the mass spectrum of SCFA isotopologues were observed, indicating that no interconversion of SCFA by the microbiota residing in the ileum took place. We hypothesize that the SCFAs are rapidly absorbed or moved distally from the sampling location in the ileum, leaving hardly a possibility for interconversion.

Although previous longer-term intervention studies suggested a role for FOS,^54^ GOS,^55^^,^^56^ or propionate^22^ in increasing plasma gut hormones GLP-1 and PYY, we did not find significant postprandial effects on these hormones during the test day. Overall, we showed that all subjects increased H_2_ in breath and non-labeled plasma acetate upon NDC consumption, but we did not find NDC fermentation or SCFA production and interconversion in the distal ileum. Therefore, the breath hydrogen increase shown here indicated most likely colonic NDC fermentation. We hypothesize that the short microbial exposure to NDC in this location due to the rapid transit time in the small intestine^57^ does not allow for this fermentation process to take place or be measurable in the volume sampled by the catheter tip. This can be considered an advantage, since the colon is prepared for microbial fermentation of NDC to SCFAs and gasses, because of the larger diameter and its capacity for distension with gas, in contrast to the small intestine where fermentation may lead to discomfort.^58^

### Dynamic changes in microbiota composition

In a review^59^ it was suggested that the small intestine microbiota rapidly responds to changes in the luminal environment, also demonstrated in ileostomy effluent microbiota that fluctuated from the morning to the afternoon on the same day.^52^^,^^60^ In our study, dynamic changes of the relative microbiota composition *in vivo* were found during the day. Characterizing the luminal microbiota over several hours *in vivo* is until now relatively unexplored. Two other clinical trials (using intestinal catheters) also show rapid fluctuations in the duodenum^61^ or duodenal, jejunal, and proximal ileal microbiota^48^ on the same day after ingestion of medication or a symbiotic, respectively. FOS and GOS are known to influence the fecal microbiota, as shown in human intervention trials where supplements were consumed for a longer period (i.e., weeks),^3^ or in *in vitro* models where bacteria were in contact with the substrates for up to 24–48 h.^49^^,^^62^ We are the first to measure several bacteria known to be stimulated by NDCs^3^ *in vivo* during the day, namely *Bifidobacterium*, *Lactobacillus*, *Bacteroides*, and *Streptococcus*, which did not significantly fluctuate over time. The lack of significance could be the result of the short contact period between microbiota and NDCs due to the rapid transit time. Since there was no fermentation at the sampling location, nor increased FOS or GOS bacterial targets or total bacteria numbers, we hypothesize that other factors, unrelated to the studied NDCs, play a role in explaining the changes in relative microbiota composition during the day.

The effects of the non-absorbable markers PEG-4000^63^^,^^64^ and TiO_2_^65^^,^^66^^,^^67^^,^^68^^,^^69^^,^^70^ on the microbiota are expected to be minor. Possibly at some time points we sampled more mucus, resulting in a higher fraction of mucus-related bacteria such as *Haemophilus*. Flushing infusions, as we also did during the ^13^C-SCFA delivery, through the catheter close to the sampling site could have impacted the relative microbiota profiles as shown previously while flushing saline through an ileal catheter.^71^ On the other hand, the transit of bacteria in the small intestine throughout one day can also be considered a physiological aspect. Others have shown that bacteria can also grow and survive in the small intestine,^50^ quickly utilizing small dietary compounds such as sugars,^52^ but the rapid bacteria fluctuations in this study are unlikely caused by bacteria doubling, given the known doubling times of minimally ∼25 min, but often 1 h or longer (*Bifidobacterium* and *Lactobacillus*).^72^^,^^73^^,^^74^ Moreover, the total bacteria numbers did not increase. It is therefore more likely that most of the dynamic changes are caused by the bacterial transit throughout the GI-tract via constantly swallowing (viable or non-viable) oral and stomach bacteria.^75^^,^^76^ Using 16S rRNA gene sequencing we could not distinguish between viable and non-viable bacteria. We hypothesize that fast transit of intestinal content rather than the studied NDCs, plays a role in explaining the changes in relative microbiota composition during the day. A reference group that did not consume NDCs was not included in our acute feeding study, making it difficult to conclude the effects of potential bacteria passage as a consequence of transit time versus the effect of the NDC bolus on the microbiota *in vivo*. The link between rapid fluctuations in the small intestine and effects on digestive processes remains to be uncovered and our findings demonstrate the relevance of sampling over time to track acute responses of the microbial community toward interventions.

### Host metabolism of luminal SCFA

To understand the mechanism by which SCFA regulate host metabolism, we studied their fate by monitoring the incorporation of ^13^C-label from the delivered SCFA into different metabolites from carbohydrates (glucose, organic acids), amino acids, and lipids (fatty acids, acyl-carnitines) metabolism. After isotope delivery, the label from ^13^C-SCFA was readily incorporated into ^13^CO_2_ in breath, and blood metabolites. Previously it has been estimated in humans that almost 95% of the produced SCFAs were rapidly absorbed.^77^ The delivered TiO_2_ was also intended to be used as a marker to differentiate between the decrease of the infused isotopes by excretion or absorption. Even though absorption is different through the complete length of the intestine,^78^ in our study, independent of the delivery location, absorption happened rapidly likely at the delivery site (distal ileum, proximal colon, or distal colon). We conclude this from the fast appearance of label in other metabolites after ^13^C-SCFA delivery and the very similar profiles of incorporation among subjects, irrespective of the delivery location. ^13^C label from all three SCFA were incorporated into glucose. Gluconeogenesis starts at the conversion of oxaloacetate in the TCA cycle into phospho-*enol*-pyruvate. Acetate and butyrate enter the TCA cycle as acetyl-CoA. Both acetate and butyrate transfer the label into glucose via oxaloacetate but do not contribute to net carbons^79^ since two carbon atoms are lost as CO_2_ during the conversion of acetyl-CoA to succinate, the precursor of oxaloacetate. Our results showed that the most efficient label transfer to glucose comes from butyrate, which is in line with previous results in mice.^18^ Almost 80% of butyrate label ended in peripheral glucose. Label incorporation from acetate to glucose suggests that acetate follows a different metabolic pathway than butyrate, even though both enter the TCA cycle as acetyl-CoA. Acetate can be activated in the cytosol and further metabolized before entering the mitochondria to be oxidized in the TCA cycle,^80^ whereas butyrate directly enters the mitochondria to be oxidized into two acetyl-CoA in a single round of β-oxidation that can immediately enter the TCA cycle.^81^ Acetate had the lowest contribution to glucose enrichment with only 12% of the acetate label ending in glucose, this may be due to high dilution by endogenous acetate production besides extra-mitochondrial metabolism. For instance, in mice, ^13^C-acetate in blood is diluted nine times compared to cecum.^24^ Propionate is the only SCFA that contributes carbons to net glucose synthesis because it enters the TCA cycle at succinyl-CoA.^82^ 23% of the intestinal propionate was used as glucose substrate. Boets et al.^20^ also described propionate as the main contributor to glucose synthesis. Glucose enrichment from propionate could have taken place in the liver or, as recently described in rats, from intestinal gluconeogenesis.^83^ Glucose from intestinal gluconeogenesis can signal through the peri-portal afferent neural system to the brain promoting metabolic benefits in energy homeostasis, such as decreased body weight and better glucose control, including decreased hepatic glucose production.^83^ This could partially explain the link between fermentable NDCs and health improvement. Nevertheless, in healthy volunteers, it is not feasible to collect portal blood to differentiate between intestinal and hepatic propionate metabolism.

Organic-acid enrichment analysis showed label incorporation only from acetate and butyrate into citrate. No label incorporation was detected from propionate into any of the organic acids. Butyrate, in line with the results on glucose enrichment, was the main contributor to citrate enrichment, since the label from butyrate is transferred to citrate as a fully labeled acetyl-CoA in the TCA cycle. Untargeted amino acids analysis showed no incorporation of label, suggesting that the delivered amount of ^13^C-SCFA was either too low or SCFA are not involved in their metabolism under fasting conditions. Moreover, we did not find incorporation of label in the most abundant plasma fatty acids, namely palmitic acid (C16:0), palmitoleic acid (C16:1), stearic acid (C18:0), and oleic acid (C18:1). It has been reported that 15% of the colonic-delivered acetate ended up in palmitic acid under standardized feeding conditions in humans,^20^ which corresponded to minimal newly synthesized fatty acids. Our even lower incorporation in fatty acids compared to Boets et al.^20^ could be due to the fasted state of the volunteers during the test day, since it is known that fasting stimulates fatty-acid oxidation rather than synthesis. Our studies, in combination with previous findings,^20^^,^^84^^,^^85^^,^^86^ show that it is unlikely that acetate produced by microbes from fermentable fiber will lead to increased blood cholesterol and fatty acid levels. In this study, we measured carnitines as an estimate for fatty acid oxidation. We did not detect M^+2^ acetyl-carnitine or M^+4^ butyryl-carnitine, which might be explained by the rapid mitochondrial conversion of butyrate into acetyl-CoA that can enter the TCA cycle, as previously described. The only acyl-carnitine enrichment in blood was M^+3^ propionyl-carnitine, originating from fully labeled propionate. Since C3-carnitines are not oxidized in the β-oxidation, this enrichment only reflects an overload of mitochondrial propionate that leaves the cell as propionyl-carnitine. The rapid increase of the percentage of labeled CO_2_ in breath comes from the metabolism of all three SCFA at the same time and can be explained by direct oxidation of SCFA and subsequent oxidation of secondary metabolites, such as glucose. This rapid label incorporation could have been potentiated by the fasted state of the volunteers. In conclusion, SCFA are rapidly absorbed and metabolized by the host, independent of intestinal delivery location.

### Conclusions

We aimed to study acute fermentation kinetics of GOS and chicory FOS in the (small) intestine in humans using a naso-intestinal catheter. We are the first to show that no NDC breakdown, and subsequent no SCFA production nor bacterial cross-feeding occurred in the distal ileum of healthy humans. This also highlights the importance of considering the passage time of the bolus through the gut when performing *in vitro* fermentations. Dynamic changes in the relative microbiota composition during the day were observed. SCFA were rapidly taken up and metabolized by the host independent of the delivery location, which could explain the lack of detecting bacterial SCFA conversions. Future studies should focus on colonic NDC fermentation for a better understanding of how NDC can influence host health to improve dietary intervention.

### Limitations of the study

Due to the high number of drop-outs because of catheter placement in the NDC intervention group in study 2, we were not able to detect an effect of the 7-day FOS:GOS intervention on the acute fermentation kinetics. A limitation of our study is that some of the measured outcomes could only be determined in a relatively low number of participants, which was attributed primarily to high drop-out rates. Consequently, there is a possibility that the effects of the NDC bolus on the changes in our outcome measurements over time, such as the gut hormones, during the test day might not have been fully captured. In most subjects, the catheter was placed in the distal ileum, and, as expected,^8^^,^^48^ the distal ileum microbiota composition and predicted functionality were distinct from those in the colon. Standardization of the sampling site in the intestine amongst subjects is therefore crucial for interpretation and comparison of the study outcomes between people, but standardizing the catheter position was challenging. We expect that a longer progression period in combination with regular checks using fluoroscopy and contrast liquid will improve positioning and standardizing of the catheter location in the proximal colon. We only used contrast liquid at the end of the test day, because it could affect some of the outcome measurements. Sampling in the fasted state (both ileum and colon) and the colon through the 1.9 mm-aspiration channel in the catheter was not feasible. The intestinal contents were obtained from a localized area of the intestine (aspiration holes within 10 cm of the tip of the catheter), providing local information on NDC fermentation and SCFA kinetics. To estimate the volume of the sampling location, we relied on the measurements of the dilution of the delivered TiO_2_. However, this non-absorbable marker was sensitive to the luminal matrix, precipitating and forming a colloidal, nonuniform intestinal liquid. Overall, the use of the naso-intestinal catheter had disadvantages, but at the moment it is one of the few available sampling tools to study the intestinal lumen in humans. We would like to note the high habitual fiber intake of the participants, when compared to the general Dutch adult population, that may affect the generalizability of our findings. Although greater NDC intervention effects are anticipated in those with lower fiber intake, we did not investigate the impact of the 7-day supplementation on the intestinal microbiota. Furthermore, the study lacked statistical power to draw conclusions about the effect of the 7-day NDC supplementation on postprandial FOS and GOS fermentation and degradation kinetics. Nevertheless, we have provided novel data about the kinetics of NDCs and the fate of SCFAs in humans as well as technical challenges to be considered when conducting *in vivo* studies in the human intestine.

## STAR★Methods

### Key resources table

**Table:** 

REAGENT or RESOURCE	SOURCE	IDENTIFIER
**Antibodies**		

rat5M-PABM-A; anti-PEG coating antibody	Institute of Biomedical Sciences, Academia Sinica, Taipei, Taiwan, ROC	rat5M-PABM-A
6.3-PABG-B biotin; anti-PEG detection antibody	Institute of Biomedical Sciences, Academia Sinica, Taipei, Taiwan, ROC	6.3-PABG-B

**Chemicals, peptides, and recombinant proteins**		

chicory FOS	Frutalose OFP; Sensus, Roosendaal, the Netherlands	NA
Vivinal GOS	FrieslandCampina Research and Development, Wageningen, the Netherlands	NA
Maltodextrin Paselli MD12	Avebe, Veendam, the Netherlands	
polyethylene glycol 4000 (macrogol)	Dulcosoft, Sanofi Aventis Nederland BV, Gouda, the Netherlands	8711642014387 (EAN-code)
TiO2 (E-171)	BrandNewCake, Baktotaal, Goor, the Netherlands	NA
Calibration gas 149 ppm H2, 74 ppm CH4, 6.1% CO2	QuinTron Instrument Company, Inc., Milwaukee, WI, USA	QT07500-G
DPP IV Inhibitor	Merck Millipore, Darmstadt, Germany	DPP4-010
Na-[1-13C1]-acetate	IsoLife B.V., Wageningen, the Netherlands	NA
Na-[1,2,3,4-13C4]-butyrate	IsoLife B.V., Wageningen, the Netherlands	NA
Na-[1,2,3-13C3]-propionate	IsoLife B.V., Wageningen, the Netherlands	NA

**Critical commercial assays**		

Total GLP-1 U-plex ELISA	MesoScale Discovery, Gaithersburg, MD, USA	K15274K
Total PYY U-plex ELISA	MesoScale Discovery, Gaithersburg, MD, USA	K15274K

**Oligonucleotides**		

Primers microbiota sequencing preparations: 515FY: 5'-GTGYCAGCMGCCGCGGTAA-3’ 806RB: 5'-GGACTACNVGGGTWTCTAAT-3’	Parada et al.^25^; Apprill et al.^26^	
Primers 16S rRNA gene copies: F: 5'-CCATGAAGTCGGAATCGCTAG-3’ R: 5'-GCTTGACGGGCGGTGT-3’	Ramseier et al.^27^	

**Software and algorithms**		

R Version 4.0.3	R: A language and environment for statistical computing. R Foundation for Statistical Computing, Vienna, Austria	https://www.r-project.org/
Chromeleon Version 7.2 SR4	Thermo Fisher Scientific, Waltham, MA, USA	
PICRUSt2	The Huttenhower Lab	https://github.com/picrust/picrust2/wiki/
QuantaSoft software Version 1.7.4.0917	Bio-Rad Laboratories, Hercules, CA, USA	

### Resource availability

#### Lead contact

Further information and requests for resources and reagents should be directed to and will be fulfilled by the Lead Contact, Guido Hooiveld (guido.hooiveld@wur.nl).

#### Materials availability

This study did not generate new unique reagents.

#### Data and code availability

• Adjective data reported in this paper will be shared by the lead contact upon request.
• This paper does not report original code.
• Any additional information required to reanalyze the data reported in this paper is available from the lead contact upon request.

### Experimental model and study participant details

In both trials, healthy male subjects with an age between 18-60 years, a BMI between 18.5-30 kg/m^2^, and regular bowel movements (defecation on average once a day) were included. Since only males were included, no sex- and gender-based analyses were performed, which might be considered a limitation to this research’s generalizability. The main exclusion criteria were having a history of medical or surgical events, the use of any prescribed or non-prescribed medication during the three weeks before study start, smoking, use of pro- pre- or antibiotics within 3 months before the study start, having infrequent bowel movements (less than three times per week), and alcohol consumption over 21 consumptions per week. Details on the participant profile can be found in Table 1. All subjects filled in a validated food frequency questionnaire during screening to determine habitual fiber intake. All subjects gave written informed consent. The studies were approved by the Medical Ethics Committee of Wageningen University.

### Method details

#### Study designs and intervention products

##### Study 1: To test a novel approach to study acute fermentation kinetics

Study 1 consisted of two days in total. On day one, subjects were intubated with a naso-intestinal catheter (Figure S1, Mui Scientific, Ontario, Canada) that progressed during the day towards the distal small intestine. In the evening a standardized meal was consumed (540 gram in total, 131 kcal/100 g, 8.6 g fat/100 g, 7.8 g carbohydrates/100 g, 4.8 g protein/100 g, 1.1 g fiber/100 g). On day two, after an overnight fast, the experimental test day took place.

##### Study 2: To study the effects of an NDC intervention on acute fermentation kinetics

Study 2 was designed as an explorative randomized, double-blind, placebo-controlled, parallel-group study. Randomization was performed to assign participants to the placebo or the NDC intervention arm. First, pairs were matched based on a similar BMI and age. An independent person randomly allocated within one couple one person to NDC and the other to placebo using a computerized procedure. All study participants and investigators were blinded to intervention allocations until all analyses were completed. Participants in the NDC group received a mixture of 7.5 g/day of chicory FOS (synonym oligofructose, Frutalose® OFP; Sensus, Roosendaal, the Netherlands) and 7.5 g/day GOS (Vivinal GOS, FrieslandCampina, Wageningen, the Netherlands) for seven days. Participants in the placebo group received isocaloric maltodextrin (13.2 g/day, Paselli MD 12, Avebe, Veendam, the Netherlands) for seven days. The NDCs were packed in closed, non-transparent jars. Subjects were asked to ingest the supplements twice daily (7.5 g mixed FOS/GOS per dose), with breakfast in the morning and with dinner in the evening. The empty and remaining jars were returned to assess compliance. Subjects were instructed to maintain their habitual diet during the study. On day seven, a fecal sample was collected immediately after defecation at home using a “FecesCatcher” (Tag Hemi, Zeijen, the Netherlands), stored at −20°C for maximally 24 hours, and afterwards stored at –80°C. Next, identical to study 1, subjects were equipped with a naso-intestinal catheter in the hospital (Gelderse Vallei, Ede, the Netherlands). In the evening, a standardized meal was consumed (380 gram in total, 137 kcal/100 g, 4.7 g fat/100 g, 17.4 g carbohydrates/100 g, 6.9 g protein/100 g, 1.4 g fiber/100 g). On day eight, after an overnight fast, the experimental test day took place.

#### Design and placement of the naso-intestinal catheter

A naso-intestinal catheter was used as an intestinal sampling and delivery tool.^28^ A custom-made 300 cm long, silicone multi-channel naso-intestinal catheter with an outer diameter of 3.5 mm, a 0.4 mm delivery channel, and a 1.9 mm aspiration channel was used (Figure S1, Mui Scientific, Ontario, Canada). The aspiration channel contained three side holes with 3-cm interspacing between each side hole (at position 1, 4, 7 cm). An inflatable balloon and three small weights were located at the tip end, and a radio-opaque marker was present for visualization by fluoroscopy. On day one (study 1) or day seven (study 2) between 07.30-09.30 h, subjects were intubated with a custom-made intestinal catheter positioned as distally as possible in the catheter. After manual placement past the ligament of Treitz using fluoroscopy, the balloon was kept inflated with 5 cc air and inserted by the subjects themselves with a maximum of 10 cm/hour. Freeze-frame fluoroscopy was applied during intubation of the duodenum, and afterwards for verification of the location at three moments. In study 2 only, the subjects were requested to set a wake-up call the morning of the experimental test day (day 8) to reconvene with the catheter progression protocol.

#### The experimental test day to study acute fermentation kinetics

After an overnight fast, subjects returned to the hospital for the test day. Before the experiment started, the location of the catheter was verified with fluoroscopy. When the position of the catheter was estimated to be in the distal ileum or colon, the experimental procedures started. After taking baseline samples of breath, blood, and intestinal content (the latter when possible), the subjects consumed a NDC bolus (assumed t=0 min). The NDC bolus consisted of 5 gram chicory FOS (Frutalose OFP; Sensus), 5 gram GOS (Vivinal GOS, FrieslandCampina), and 5 gram of non-digestible marker polyethylene glycol 4000 (PEG 4000 gram/mol) (Dulcosoft, Sanofi-Aventis, the Netherlands) in 200 mL tap water. GOS contained ≤10 bacteria colony-forming units (CFU)/gram mixture, and FOS contained ≤100 CFU/gram mixture. Subjects were not allowed to eat or drink during measurements, except for (tap) water. In the period the study took place, tap water in the hospital contained on average <1 CFU/mL, and maximally 5 CFU/mL (Vitens Laboratory, Leeuwarden, the Netherlands). The NDC bolus contained maximally 1550 CFU, and the intra-intestinal infusion contained ≤15 CFU (measured as 16S rRNA gene copies). After NDC bolus consumption, ^13^C-labeled SCFA were directly delivered into the intestinal lumen via the catheter delivery channel, at 145 minutes in study 1 and 125 minutes in study 2 respectively. The ^13^C-SCFA infusion consisted of 0.96 M Na-[1-^13^C_1_]-acetate, 0.40 M Na-[1,2,3-^13^C_3_]-propionate, and 0.09 M Na-[1,2,3,4-^13^C_4_]-butyrate, all with an isotopic purity over 99% (IsoLife B.V., Wageningen, the Netherlands), in 10 mL ultrapure water (Merck Millipore, Germany). After delivery, 100 mg non-digestible absorption marker TiO_2_ (E-171, BrandNewCake, Baktotaal, Goor, the Netherlands) in 10 mL (study 1) or 5 mL (study 2) of ultrapure water was delivered. Both ^13^C-SCFA and TiO_2_ solutions were delivered via the delivery channel of the naso-intestinal catheter. During the test day, blood, breath, and intestinal content were collected at multiple time points. The time points and frequency of sampling differed slightly between study 1 and study 2, as study 1 had 100 minutes longer test day (Figure 1). At the end of the test day, the exact location of the catheter was determined using 50 mL contrast liquid (Telebrix GASTRO, Guerbet, Aulnay-sous-Bois, France, diluted 1:1 with water) and fluoroscopy. In study 2 subjects were asked to indicate the intensity of (dis)comfort caused by the study procedures by marking a 100-mm-long horizontal line, visual analog scales (VAS), that is labeled with ‘no pain/discomfort’ at the 0 mm end and ‘a lot of pain/discomfort’ at the 100 mm end.

#### Sample collection

Breath samples were collected in collection bags (QuinTron instrument company, Milwaukee, USA) that were closed with a stopcock. Luminal samples were collected in 5 mL tubes, homogenized, and divided into aliquots which were put on dry ice immediately, and at the end of the test day stored at -80°C. The time to take ∼2.0 mL luminal sample through the aspiration channel was monitored per time point during the complete experimental test day. Blood samples (4.5 mL per time point) were collected from an indwelling venous cannula (IVC) in the left arm vena in lithium heparin tubes for SCFA and metabolite (^13^C enrichment) analysis.^29^ In study 2, additionally, 4 mL blood per time point was collected in tubes coated with potassium EDTA and aprotinin, pre-filled with 44 μL DPP IV Inhibitor (Merck Millipore), and kept on ice, for analysis of gut hormones. All blood tubes were put on ice immediately after blood withdrawal, inverted 10 times, and centrifuged (10 min, 1200xg, 4°C). The plasma was collected and divided into aliquots. All aliquots of luminal and plasma samples were kept on dry ice during the experimental test day, and afterwards stored at -80°C.

#### Analysis of breath samples

H_2_ and CH_4_ parts per million (ppm) in the exhaled breath were analyzed in a BreathTracker DP (QuinTron), according to the manufacturer’s instructions. The BreathTracker was calibrated with calibration gas with known composition (149 ppm H_2_, 74 ppm CH_4_, 6.1% CO_2_, QuinTron). To measure ^13^CO_2_ enrichment in breath, samples were transferred (flushed in excess) from the collection bags to 12 mL glass tubes with lid, and measured in a GC (Agilent Technologies, 7890A GC system), coupled to an IRMS (Thermo Scientific, Delta5Advantage) with helium as a carrier gas, as described.^30^ CO_2_ was separated from nitrogen and oxygen on a Chrompack PLOT fused silica 25mx0.25 mm ID, coated with Poraplot Q (DF = 8 μm). Water was removed after column separation by Nafion tubing. The isotopologue spectra were recorded at m/z:44 and m/z:45 for ^12^CO_2_ and ^13^CO_2_, respectively, and corrected for ^17^O.

#### Analysis of luminal content

##### Analysis of NDC in the luminal content

Luminal samples were analyzed for mono-, di-, and oligosaccharide profiles by ICS 3000 high performance anion exchange chromatography (HPAEC, Dionex Corporation, Sunnyvale, CA, USA) with pulsed amperometric detection (PAD).^31^ 100 μL luminal content was centrifuged (10 min, 4°C, 15 000xg), and the supernatant was diluted (10-300 times). 20 μL was injected into the system, where compounds were separated on a CarboPac PA-1 guard column (2 × 50 mm) followed by a CarboPac PA-1 column (2 × 250 mm). The elution gradient consisted of first a linear gradient of 0.02–0.05 M NaOH in 3 min and 0.05–0.075 M NaOH in 10 min, followed by isocratic elution of 0.1 M NaOH for 2 min, and a 50-minute gradient of 0–1 M NaOAc in 0.1 M NaOH, with a flow rate of 0.3 mL/min.^32^ Identification of FOS and GOS isomers was based on commercial standards, and the elution profiles of the luminal content were compared with the elution profiles of FOS, Vivinal GOS, and its DP fractions, and to GOS and FOS profiles obtained in previous research.^33^^,^^34^ Data were analyzed with Chromeleon 7.2 SR4 software.

##### Microbiota composition, predicted functionality and 16S rRNA gene copy numbers in the luminal content

50-250 μL of intestinal sample or 150 mg feces was used for the analysis of the microbiota composition, as described in,^31^ with minor changes. After lysis of the cells in the luminal samples, the obtained lysate (around 500 μL) was divided into two aliquots and used separately for DNA isolation, DNA of both aliquots was collected in the same elution tube within 30 μL nuclease-free water. All details of the PCR amplification, purification, quantification, and pooling are described elsewhere.^31^ Each PCR reaction contained 7 μL 5 × Phusion Green HF buffer, 0.7 μL 10 mM dNTPs (Promega, Madison, USA), 0.4 μL Phusion hot start II DNA polymerase (2 U/μL), 25.5 μL nuclease-free water, 0.7 μL of 20 ng/μL DNA and 0.7 μL of each of the barcoded primers 515F^25^ and 806R^26^ (10 μM). Cycling conditions were as follows: 98°C 30 s, 25 cycles of 98°C 10 s, 50°C 10 s, 72°C 10 s, and 72°C for 7 min. The PCR products were purified using magnetic beads (MagBio Genomics Inc., Gaithersburg, USA), and concentrations were measured using Qubit dsDNA BR buffer and dye (Invitrogen, California, USA), on a DS-11 FX fluorometer (DeNovix, Wilmington, USA). An equimolar mix (200 ng each) of purified PCR products was prepared. Microbiota composition was determined via sequencing of the variable V4 region of the 16S rRNA gene using Illumina HiSeq2500. Raw sequencing data were processed using NG-Tax 2.0 pipeline with default settings.^35^^,^^36^ Taxonomy was assigned using the SILVA database (version 132). Microbial functions were predicted based on the 16S rRNA gene sequences using the phylogenetic investigation of communities by reconstruction of unobserved states algorithm (version PICRUSt2) with default settings but the minimum alignment was set to 60%.^37^

The total 16S rRNA gene copy numbers were determined by digital droplet PCR (ddPCR), using universal 16S rRNA gene primers.^27^ All materials for the ddPCR analysis were ordered from Bio-Rad Laboratories, Carlsbad, CA, USA. DNA was diluted to 0.005 ng/μL. Per 1 μL diluted DNA, 19 μL master mix was added (7 μL milli-Q water, 10 μL QX200 EvaGreen ddPCR Supermix, 1 μL universal 16S rRNA gene forward and reverse primers^27^) in cartridges. The 20 μL sample with mix was pipetted perpendicular into cartridges for QX200 Droplet Generator and placed in cartridge holders. Wells without sample were filled with 1 μL water and 19 μL mastermix. 70 μL Droplet Generation Oil for EvaGreen was also added in the cartridge, covered with Droplet Generator DG8 Gaskets, and droplets were generated (QX100 Droplet Generator). Next, 40 μL of the generated droplets was transferred into a ddPCR 96-well plate, and sealed with Pierceable Foil Heat Seal in the PX1 PCR plate sealer at 170°C for 5 seconds. PCR amplification took place at 95.0°C for 10 min, 40 times 95.0°C for 30 sec and 58°C for 1 min, followed by 4°C for 5 min, and 90.0°C for 4 min. Data was generated using the QX200 Droplet Reader, and analyzed using QuantaSoft software (Version 1.7.4.0917).

##### Analysis of SCFA concentration and ^13^C enrichment in the luminal content

SCFA concentration and ^13^C-enrichment in luminal sample were analyzed as described previously.^31^ Luminal samples were thawed and 100 μL were diluted in PBS and spiked with 100 μL of (0.5 mg/mL) 2-ethyl butyric acid as internal standard. After adding 20 μL of 20% 5-sulfosalicylic acid and 10 μL of HCl, samples were homogenized by bead beating for 60 s at 6000 rpm (Precellys 24, Bertin Technologies, Montigny Le Bretonneux, France) at 4°C. After 20 min centrifugation at 15000 g at 4°C, the supernatant was transferred to a glass vial and SCFA were extracted with 2 mL diethyl ether.^31^ After 10 min vortexing and 10 min centrifugation at 1200 g at 4°C, derivatization was performed with 500 μL of the supernatant and 50 μL of N-tert-Butyldimethylsilyl-N-methyltrifluoroacetamide (MTBSTFA) overnight at room temperature. Mass spectrometry analysis was performed by electron ionization. The mass isotopologue spectra of ([M-57]^+^) fragment of the t-BDMS derivatives of acetate (m/z 117-1119, m_0_-m_2_), propionate (m/z 131-134, m_0_-m_3_), butyrate (m/z 145-149, m_0_-m_4_) and 2-ethyl butyric acid (m/z 173) were monitored.

##### Measurement of non-absorbable markers in the luminal content

Titanium dioxide (TiO_2_) in the intestinal content was analyzed in 100 μL sample. The samples were dissolved in 1000 μL water, transferred to a digestion tube, and digested with 1 mL HF and 7 mL HNO_3_ in a total volume of 50 mL water. These digested samples were analyzed by Thermo X 129 Series-2 HR-ICP-MS equipped with an autosampler, a conical glass concentric nebulizer, and 130 operated at an RF power of 1400 W, according to.^38^ The limit of quantification was 0.05 mg Ti/kg of wet sample. PEG-4000 was quantified by ELISA using “rat5M-PABM-A anti-PEG” as coating, and “6.3-PABG-B biotin anti-PEG” as detection antibodies, respectively (IBMS Academia Sinica, Taiwan), according to supplier’s instructions.

#### Analysis of plasma samples

##### Short chain fatty acid quantification in plasma

To quantify SCFA concentrations in plasma, the samples were derivatized with 3-nitrophenylhydrazine hydrochloride (3NPH-HCl) and subsequently measured by LC-MS/MS as previously described.^39^ Plasma samples were diluted 1:3 with 100% acetonitrile (ACN) on room temperature and mixed by hand, and centrifuged for 20 min at 14000xg at 21°C. The samples were derivatized with 3-nitrophenylhydrazine hydrochloride (3NPH-HCl). 50 μL of the supernatants were mixed with 50 μL 200 mM 3NPH-HCl in 50% (v/v) aqueous ACN and 50 μL 120 mM N-(3-dimethylaminopropyl)-N′-ethylcarbodiimide in 6% (v/v) pyridine in 50% (v/v) aqueous ACN solution in low binding Eppendorf tubes. After mixing, the tubes were incubated in an Eppendorf thermomixer at 40°C for 30 min, afterwards placed on ice for at least 1 min and further diluted with 100 μL milli-Q water. 90 μL of the derivatized sample was transferred to a UPLC vial containing a 10 μL internal standard mix containing 125 μM acetate-^13^C-3NPH, 100 μM propionate-^13^C-3NPH and 100 μM butyrate-^13^C-3NPH. Samples were measured by LC-MS/MS using a Shimadzu LCMS-8050 triple quadruple mass spectrometer (Kyoto, Japan). SCFA concentrations were quantified using an external calibration curve constructed under the same conditions as the plasma samples. All plasma samples were derivatized and measured.

##### Glucose ^13^C enrichment and concentration in plasma

Glucose ^13^C enrichment was measured by GC/MS using a penta-acetate derivative according to Van Dijk et al..^40^ To 50 μL of plasma 500 μL ice-cold ethanol was added and samples were kept on ice for 45 min. Subsequently, samples were centrifuged (10 min, RT, 20,000xg), and 200 μL supernatant was transferred to a Teflon-capped reaction tube and dried at 60°C under a stream of nitrogen. After cooling to room temperature, 100 μL pyridine and 200 μL acetic anhydride were added to the samples, incubated for 30 minutes at 60°C, and subsequently dried at 60°C under a stream of nitrogen. Finally, the residues were dissolved in 200 μL ethylacetate and transferred into GC injection vials with insert. Ions monitored were m/z 408-414 (m_0_-m_6_). Total glucose concentrations were measured in 10 μL plasma by using a blood glucose meter (Accu Check Performa) and test strips (Roche, Indiana, USA).

##### Organic and amino acids concentrations and ^13^C enrichments in plasma

Organic and amino acids concentrations and ^13^C enrichments were quantified as described by Evers et al.^41^ To 100 μL of plasma in a glass tube 900 μL of milli-Q water was added. Next, on ice, 2 mL ice-cold chloroform was added, the tubes were closed and vortexed for 30 min at 4°C. After, samples were centrifuged at 1250xg for 10 min. The upper aqueous phase was transferred to a clean glass tube and dried at 37°C under a stream of nitrogen. The sample was derivatized by the addition of 40 μL methoxyamine.HCL in pyridine (2% v/v), incubated for 90 min at 37°C, cooled down at RT, centrifuged for 1 min at 1250xg at RT, after which 60 μL of MBTSTFA + 1% TBDMCS was added, incubated at 55°C for 60 min and cooled down at RT. Finally, all tubes were centrifuged for 10 min at 1250xg RT, and the derivatized sample was transferred into a glass vial with an insert with a screw-cap for GC-MS analysis.

##### Measurement of free carnitine and acylcarnitines concentrations and ^13^C enrichments in plasma

Free carnitine and acyl-carnitines were measured according to Derks et al.^42^ For carnitine analysis the samples were prepared for concentration and ^13^C enrichments. For concentration: 100 μL of cold acetonitrile was added to 10 μL of plasma sample, shortly vortexed and then 100 μL of internal standard ([8,8,8-^2^H_3_]-octanoyl-L-carnitine and [10,10,10-^2^H_3_]-decanoyl-L-carnitine) was also added to the mix. For enrichments: 100 μL of cold acetonitrile was added to 10 μL of plasma sample, shortly vortexed, and then 100 μL of 80% methanol in milli-Q water was added to the mix. After, samples from both sets were vortexed again and centrifuged (RT, max speed 20,000g) for 10 min. Finally, 150 μL from each sample were transferred to a GC injection vial with insert for analysis.

###### Fatty acids ^13^C enrichments in plasma

Fatty acids ^13^C enrichments were measured as described by Muskiet et al.^43^ To 50 μL of plasma, C17 internal standard (50 mg C17:0 in 100 mL Methanol) were added. Fatty acids were hydrolyzed in 2 mL of methanol-HCl (5:1 vol/vol) for 4 hours at 90°C in a closed glass tubes. Fatty acids were later extracted in 2 mL hexane, vortexed, centrifuged (5 min at 800xg) and evaporated while heating at 45°C. The methylated fatty acids were re-dissolved in 200 μL hexane and pipette into a new GC-vial with insert to be analyzed.

##### GLP-1 and PYY concentrations in plasma

Total GLP-1 and total PYY concentrations were measured in 50 μL of undiluted plasma, using the multiplex sandwich immunoassay system from MesoScale Discovery (Gaithersburg, MD, USA) according to manufacturer’s instructions on a MESO QuickPlex SQ 120MM system (MesoScale Discovery). The intra-assay CV of the total GLP-1 assay was 8.4%, the intra-assay CV of the total PYY assay was 9.1%.

#### Corrections and calculations

##### Normalization of the mass isotopologues distributions measured by GC-MS

All data from plasma samples measured by GC-MS (m0-m+6) was first corrected for the natural abundance of ^13^C by multiple linear regression according to Lee et al.^44^ To obtain the excess fractional distribution of mass isotopologues (M^0^-M^+6^). Data from luminal samples were not corrected by natural abundance since there were technical issues to evaluate quantitative all different label patterns. However, the results are shown as percentage of the label and clearly described the lack of measurable production and interconversion of SCFA.

##### Acetate, propionate, and butyrate contribution to blood metabolites

The individual contributions of acetate, propionate, and butyrate to the labeling of plasma metabolites were calculated by taking into consideration known biochemical pathways, the stoichiometry to molecules of plasma metabolite per each SCFA, and the amount delivered (all data was normalized to the delivered acetate concentration to compare for efficiency of individual SCFA). For glucose, ^13^C carbons in [1,2,3-^13^C_3_]-propionate will enter the TCA cycle as succinate and will be scrambled when converted to fumarate resulting in [1,2,3-^13^C_3_]-oxaloacetate and [2,3,4-^13^C_3_]-oxaloacetate, respectively. The conversion of these oxaloacetate isotopologues into phosphoenolpyruvate (PEP) by the PEP carboxykinase (PEPCK) will result in [1,2,3-^13^C_3_]-PEP and [2,3-^13^C_2_]-PEP, respectively, and equal contribution of M^+3^ and M^+2^ glucose to the isotopologue distribution, respectively. The mitochondrial breakdown of [1,2,3,4-^13^C_4_]-butyrate to two [1,2-^13^C_2_]-acetyl-CoA and subsequent reaction with oxaloacetate to [4,5-^13^C_2_]-citrate entering the TCA cycle leads to equal contribution of M^+1^ and M^+2^ glucose due to scrambling at fumarate in the TCA cycle. Moreover, 1 molecule of butyrate gives rise to 2 molecules of acetate labeling 2 molecules of glucose. [1-^13^C_1_]-acetate is converted to [1-^13^C_1_]-acetyl-CoA equally contributing to M^+0^ and M^+1^ glucose to the isotopologue distribution due to scrambling at fumarate in the TCA cycle. In the case of citrate, as described for glucose, the breakdown of [1,2,3,4-^13^C_4_]-butyrate leads to two [1,2-^13^C_2_]-acetyl-CoA which in turn leads to 2 [1,2-^13^C_2_]-citrate molecules in the TCA cycle. [1-^13^C_1_]-acetate leads to [1-^13^C_1_]-acetyl-CoA leading to 1 molecule of [1-^13^C_2_]-citrate. Thus, butyrate contributes to M^+2^ and acetate to the M^+1^ citrate enrichment. Multiple linear regression was performed with the measured and corrected isotopologue distribution of glucose and citrate and the expected labeling by the SCFA.

#### Kinetics and parameter estimation of 13C-SCFA incorporation into glucose calculated by a three compartment computational model

##### Model strategy

To derive the kinetic constants for the incorporation of carbons from each SCFA into glucose, we constructed a three-compartment model, consisting of the gut, the liver and the blood compartments (Figure 8). The pool sizes in the gut were denoted as A_gut_ for acetate, P_gut_ for propionate and B_gut_ for butyrate, and expressed in mmol. The initial pool sizes of the tracers in the gut were 9.6 mmoles of acetate, 4 mmoles of propionate and 0.9 mmol of butyrate. These initial values refer to the ^13^C-SCFA delivered directly into the gut lumen through the catheter. The pool of labelled acetate in the liver, denoted as A_liver_ and also expressed in mmol, had an initial value of 0 mmol. The plasma pools of ^13^C-glucose derived from acetate, propionate and butyrate were expressed in mM and denoted by G_a_, G_p_ and G_b_, respectively. The different units for gut and liver (mmol) on the one hand and plasma (mM) on the other hand were inspired by the experimental design: the delivered amount of tracer was known in mmol, whereas the glucose concentration was measured in mM. To relate these units to each other, the volume of the plasma compartment was 0.15 L of blood per kg bodyweight.^45^ The total plasma volume, taking into account the body weight of each subject, was denoted as V_plasma_ and is expressed in L. The apparent rates constants, expressed in min^-1^, are:

k_1a_ : rate constants for uptake of acetate from gut to liver

k_1p_ : lumped rate constant of uptake of propionate from the gut and conversion to glucose delivered to the plasma

k_1b_ : lumped rate constant of uptake of butyrate from the gut and conversion to glucose delivered to the plasma

k_La_: lumped rate constant of conversion of acetate to glucose in the liver and delivery to the plasma

k_2_ : rate constant of disappearance of labeled glucose from the plasma, with the same value irrespective of the SCFA from which the label was derived.

k_0a_: rate constant of loss of acetate to other fates than glucose

k_0p_: rate constant of loss of propionate to other fates than glucose

k_0b_: rate constant of loss of butyrate to other fates than glucose

This led to the following system of ordinary differential equations.

$$\frac{dA_{gut}}{dt}=-\left(k_{1a}+k_{0a}\right)*A_{pool}$$

$$\frac{dA_{Liver}}{dt}=k_{1a}*A_{pool}-k_{La}*A_{Liver}$$

$$\frac{dG_{a}}{dt}=\frac{1}{V_{Plasma}}*\left(k_{La}*A_{Liver}-k_{2}*V_{Plasma}*G_{a}\right)$$

$$\frac{dP_{gut}}{dt}=-\left(k_{1p}+k_{0p}\right)*P_{pool}$$

$$\frac{dG_{p}}{dt}=\frac{1}{V_{Plasma}}*\left(k_{1p}*P_{pool}-k_{2}*V_{Plasma}*G_{p}\right)$$

$$\frac{dB_{gut}}{dt}=-\left(k_{1b}+k_{0b}\right)*B_{pool}$$

$$\frac{dG_{b}}{dt}=\frac{1}{V_{Plasma}}*\left(k_{1b}*B_{pool}-k_{2}*V_{Plasma}*G_{b}\right)$$

Note that the conversion factor of V plasma accounts for the fact that the glucose pools are expressed in mM and the SCFA pools in mmol.

##### Assessing identifiability of kinetic rate parameters and model fitting

In order to estimate the model parameters, they need to be identifiable. To assess this, the multivariate parameter identifiability in the R package FME was used and the collinearity index of parameter combinations was calculated.^46^ A collinearity index below 20 indicates that the parameters in the combination are identifiable. When the plasma glucose enrichment data of all subjects were averaged, the maximum number of model parameters that could be identified together was six out of the eight (Figure S8).

To estimate the six identifiable parameters, two of the parameters needed to be fixed. The first parameter selected to be fixed was k_La_, since this parameter did not have sufficient data to support a reliable prediction. The second parameter was selected as the parameter that gave the minimal collinearity if it were to be fixed together with k_La_. The values of these two parameters were estimated by grid search between 0 and 1, selecting the values that gave the smallest root mean squared error (RMSE). Based on the average data set. these values were 0.0071 and 0.0109 for k_La_ and k_0p_, respectively. The RMSE was calculated from:

$$RMSE=\sqrt{\sum_{i=1}^{n}{\left({\hat{Y}}_{i}-Y_{i}\right)}^{2}}$$

where $Y_{i}$ and ${\hat{Y}}_{i}$ denote the observed and model fitted data points for time i, respectively.

The model simulation deSolve in the R package was used.^47^ The parameters were fitted by minimizing the sum of squared differences between model simulation and the measured data points divided by the standard deviation of the measured data points in the plasma, according to:

$$SSE=\sum_{i=1}^{n}{\left(\frac{{\hat{Y}}_{i}-Y_{i}}{s}\right)}^{2}$$

###### Fractional dose contribution

This was calculated in two different ways, (i) as the fraction of rate contants: $\frac{k_{1a}}{k_{1a}+k_{0a}}$*,* $\frac{k_{1p}}{k_{1p}+k_{0p}}$*,* $\frac{k_{1b}}{k_{1b}+k_{0b}}$ and (ii) with the following pharmacodynamic formula :

$$Fraction=\frac{AUC\;\left(mmoles*\min*L^{-1}\right)*CL\;\left(L*min^{-1\;}\right)}{SCFA\;pool\;\left(mmoles\right)}$$

$$CL\;\left(clearence\right)=k_{2}\left(min^{-1}\right)*V_{plasma}$$

Provided that the AUC was calculated over the whole fitted curve, i.e. until the tracer concentration was negligible, the two methods gave the same results, as should be the case based on theoretical considerations.

### Quantification and statistical analysis

Differences in baseline characteristics between groups were evaluated with the Kruskal Wallis test. Differences in the fecal microbiota composition between groups were evaluated with the non-parametric Mann-Whitney U test, followed by a false discovery rate correction for multiple comparisons. Per parameter, normality was tested by histograms, Q-Q-plots, and the Shapiro-Wilk test of normality. If not normally distributed, the variable was log-transformed (base 10) to improve normality. A one-way repeated measures ANOVA, or mixed models when there were numerous missing values, was applied to check for changes over time during the experimental test day. For the microbiota analyses, the 16S rRNA gene counts were normalized to relative abundance. Bray–Curtis dissimilarity was calculated within each subject for the microbiota in the luminal content at each time point compared to the first sample that was collected on the test day. The alpha-diversity was calculated based on the amplicon sequence variants, using several diversity indices. Principle coordinate analysis based on weighted UniFrac was used to evaluate the overall microbiota variation at the amplicon sequence variant level (beta-diversity). To investigate changes in microbiota alpha-diversity indices or selected arcsin-square root transformed bacteria proportions over time, mixed models were used. In case of significant overall time effects, pairwise post-hoc comparisons were made. Statistical significance was accepted as P<0.05. Statistical and microbiota analyses were performed in R version 4.0.3.

### Additional resources

Prior to enrolling participants, the trials were registered at ClinicalTrials.gov, identifiers: NCT04013607 (study 1) and NCT04499183 (study 2).

## Supplemental information

Document S1. Figures S1–S16 and Table S2

Table S1. The relative abundances^1^ of the microbiota on genus level in the luminal content and the feces of healthy male subjects, related to Figure 2

## References

[1] Zhu T., Goodarzi M.O. (2020). Metabolites Linking the Gut Microbiome with Risk for Type 2 Diabetes. Curr. Nutr. Rep..

[2] Anderson J.W., Randles K.M., Kendall C.W., Jenkins D.J. (2004). Carbohydrate and fiber recommendations for individuals with diabetes: a quantitative assessment and meta-analysis of the evidence. J. Am. Coll. Nutr..

[3] Swanson K.S., de Vos W.M., Martens E.C., Gilbert J.A., Menon R.S., Soto-Vaca A., Hautvast J., Meyer P.D., Borewicz K., Vaughan E.E., Slavin J.L. (2020). Effect of fructans, prebiotics and fibres on the human gut microbiome assessed by 16S rRNA-based approaches: a review. Benef. Microbes.

[4] Flint H.J., Scott K.P., Duncan S.H., Louis P., Forano E. (2012). Microbial degradation of complex carbohydrates in the gut. Gut Microb..

[5] Flint H.J., Duncan S.H., Scott K.P., Louis P. (2007). Interactions and competition within the microbial community of the human colon: links between diet and health. Environ. Microbiol..

[6] den Besten G., van Eunen K., Groen A.K., Venema K., Reijngoud D.J., Bakker B.M. (2013). The role of short-chain fatty acids in the interplay between diet, gut microbiota, and host energy metabolism. J. Lipid Res..

[7] Tan J., McKenzie C., Potamitis M., Thorburn A.N., Mackay C.R., Macia L. (2014). The role of short-chain fatty acids in health and disease. Adv. Immunol..

[8] Vasapolli R., Schütte K., Schulz C., Vital M., Schomburg D., Pieper D.H., Vilchez-Vargas R., Malfertheiner P. (2019). Analysis of Transcriptionally Active Bacteria Throughout the Gastrointestinal Tract of Healthy Individuals. Gastroenterology.

[9] Zmora N., Zilberman-Schapira G., Suez J., Mor U., Dori-Bachash M., Bashiardes S., Kotler E., Zur M., Regev-Lehavi D., Brik R.B.-Z. (2018). Personalized Gut Mucosal Colonization Resistance to Empiric Probiotics Is Associated with Unique Host and Microbiome Features. Cell.

[10] Wong J.M.W., de Souza R., Kendall C.W.C., Emam A., Jenkins D.J.A. (2006). Colonic health: fermentation and short chain fatty acids. J. Clin. Gastroenterol..

[11] Minekus M., Smeets-Peeters M., Bernalier A., Marol-Bonnin S., Havenaar R., Marteau P., Alric M., Fonty G., Huis in't Veld J.H. (1999). A computer-controlled system to simulate conditions of the large intestine with peristaltic mixing, water absorption and absorption of fermentation products. Appl. Microbiol. Biotechnol..

[12] Blaak E.E., Canfora E.E., Theis S., Frost G., Groen A.K., Mithieux G., Nauta A., Scott K., Stahl B., van Harsselaar J. (2020). Short chain fatty acids in human gut and metabolic health. Benef. Microbes.

[13] Deroover L., Verspreet J., Luypaerts A., Vandermeulen G., Courtin C.M., Verbeke K. (2017). Wheat Bran Does Not Affect Postprandial Plasma Short-Chain Fatty Acids from (13)C-inulin Fermentation in Healthy Subjects. Nutrients.

[14] Lund E.K., Johnson I.T. (1991). Fermentable carbohydrate reaching the colon after ingestion of oats in humans. J. Nutr..

[15] Boll E.V.J., Ekström L.M.N.K., Courtin C.M., Delcour J.A., Nilsson A.C., Björck I.M.E., Östman E.M. (2016). Effects of wheat bran extract rich in arabinoxylan oligosaccharides and resistant starch on overnight glucose tolerance and markers of gut fermentation in healthy young adults. Eur. J. Nutr..

[16] Ibrügger S., Vigsnæs L.K., Blennow A., Skuflić D., Raben A., Lauritzen L., Kristensen M. (2014). Second meal effect on appetite and fermentation of wholegrain rye foods. Appetite.

[17] Fernandes J., Vogt J., Wolever T.M.S. (2011). Inulin increases short-term markers for colonic fermentation similarly in healthy and hyperinsulinaemic humans. Eur. J. Clin. Nutr..

[18] den Besten G., Havinga R., Bleeker A., Rao S., Gerding A., van Eunen K., Groen A.K., Reijngoud D.-J., Bakker B.M. (2014). The short-chain fatty acid uptake fluxes by mice on a guar gum supplemented diet associate with amelioration of major biomarkers of the metabolic syndrome. PLoS One.

[19] Boets E., Deroover L., Houben E., Vermeulen K., Gomand S.V., Delcour J.A., Verbeke K. (2015). Quantification of in Vivo Colonic Short Chain Fatty Acid Production from Inulin. Nutrients.

[20] Boets E., Gomand S.V., Deroover L., Preston T., Vermeulen K., De Preter V., Hamer H.M., Van den Mooter G., De Vuyst L., Courtin C.M. (2017). Systemic availability and metabolism of colonic-derived short-chain fatty acids in healthy subjects: a stable isotope study. J. Physiol..

[21] Neis E.P., van Eijk H.M., Lenaerts K., Olde Damink S.W., Blaak E.E., Dejong C.H., Rensen S.S. (2019). Distal versus proximal intestinal short-chain fatty acid release in man. Gut.

[22] Chambers E.S., Viardot A., Psichas A., Morrison D.J., Murphy K.G., Zac-Varghese S.E.K., MacDougall K., Preston T., Tedford C., Finlayson G.S. (2015). Effects of targeted delivery of propionate to the human colon on appetite regulation, body weight maintenance and adiposity in overweight adults. Gut.

[23] Canfora E.E., Jocken J.W., Blaak E.E. (2015). Short-chain fatty acids in control of body weight and insulin sensitivity. Nat. Rev. Endocrinol..

[24] den Besten G., Lange K., Havinga R., van Dijk T.H., Gerding A., van Eunen K., Müller M., Groen A.K., Hooiveld G.J., Bakker B.M., Reijngoud D.J. (2013). Gut-derived short-chain fatty acids are vividly assimilated into host carbohydrates and lipids. Am. J. Physiol. Gastrointest. Liver Physiol..

[25] Parada A.E., Needham D.M., Fuhrman J.A. (2016). Every base matters: assessing small subunit rRNA primers for marine microbiomes with mock communities, time series and global field samples. Environ. Microbiol..

[26] Apprill A., McNally S., Parsons R., Weber L. (2015). Minor revision to V4 region SSU rRNA 806R gene primer greatly increases detection of SAR11 bacterioplankton. Aquat. Microb. Ecol..

[27] Ramseier C.A., Kinney J.S., Herr A.E., Braun T., Sugai J.V., Shelburne C.A., Rayburn L.A., Tran H.M., Singh A.K., Giannobile W.V. (2009). Identification of pathogen and host-response markers correlated with periodontal disease. J. Periodontol..

[28] Ph van Trijp M., Wilms E., Ríos-Morales M., Masclee A.A., Brummer R.J., Witteman B.J., Troost F.J., Hooiveld G.J. (2021). Using naso- and oro-intestinal catheters in physiological research for intestinal delivery and sampling *in vivo*: practical and technical aspects to be considered. Am. J. Clin. Nutr..

[29] Deroover L., Boets E., Tie Y., Vandermeulen G., Verbeke K. (2017).

[30] Patel S.K., Pratap C.B., Jain A.K., Gulati A.K., Nath G. (2014). Diagnosis of Helicobacter pylori: what should be the gold standard?. World J. Gastroenterol..

[31] Rios-Morales M., van Trijp M.P.H., Rösch C., An R., Boer T., Gerding A., de Ruiter N., Koehorst M., Heiner-Fokkema M.R., Schols H.A. (2021). A toolbox for the comprehensive analysis of small volume human intestinal samples that can be used with gastrointestinal sampling capsules. Sci. Rep..

[32] Jonathan M.C., van den Borne J.J., van Wiechen P., Souza da Silva C., Schols H.A., Gruppen H. (2012). In vitro fermentation of 12 dietary fibres by faecal inoculum from pigs and humans. Food Chem..

[33] van Leeuwen S.S., Kuipers B.J.H., Dijkhuizen L., Kamerling J.P. (2016). Comparative structural characterization of 7 commercial galacto-oligosaccharide (GOS) products. Carbohydr. Res..

[34] Akbari P., Fink-Gremmels J., Willems R.H.A.M., Difilippo E., Schols H.A., Schoterman M.H.C., Garssen J., Braber S. (2017). Characterizing microbiota-independent effects of oligosaccharides on intestinal epithelial cells: insight into the role of structure and size: Structure-activity relationships of non-digestible oligosaccharides. Eur. J. Nutr..

[35] Poncheewin W., Hermes G.D.A., van Dam J.C.J., Koehorst J.J., Smidt H., Schaap P.J. (2019). NG-Tax 2.0: A Semantic Framework for High-Throughput Amplicon Analysis. Front. Genet..

[36] Ramiro-Garcia J., Hermes G., Giatsis C., Sipkema D., Zoetendal E., Schaap P., Smidt H. (2018). NG-Tax, a highly accurate and validated pipeline for analysis of 16S rRNA amplicons from complex biomes. F1000Research.

[37] Langille M.G.I., Zaneveld J., Caporaso J.G., McDonald D., Knights D., Reyes J.A., Clemente J.C., Burkepile D.E., Vega Thurber R.L., Knight R. (2013). Predictive functional profiling of microbial communities using 16S rRNA marker gene sequences. Nat. Biotechnol..

[38] Peters R.J.B., van Bemmel G., Herrera-Rivera Z., Helsper H.P.F.G., Marvin H.J.P., Weigel S., Tromp P.C., Oomen A.G., Rietveld A.G., Bouwmeester H. (2014). Characterization of Titanium Dioxide Nanoparticles in Food Products: Analytical Methods To Define Nanoparticles. J. Agric. Food Chem..

[39] van Dongen K.C.W., van der Zande M., Bruyneel B., Vervoort J.J.M., Rietjens I.M.C.M., Belzer C., Beekmann K. (2021). An *in vitro* model for microbial fructoselysine degradation shows substantial interindividual differences in metabolic capacities of human fecal slurries. Toxicol. Vitro.

[40] van Dijk T.H., Boer T.S., Havinga R., Stellaard F., Kuipers F., Reijngoud D.J. (2003). Quantification of hepatic carbohydrate metabolism in conscious mice using serial blood and urine spots. Anal. Biochem..

[41] Evers B., Gerding A., Boer T., Heiner-Fokkema M.R., Jalving M., Wahl S.A., Reijngoud D.-J., Bakker B.M. (2021). Simultaneous quantification of the concentration and carbon isotopologue distribution of polar metabolites in a single analysis by gas chromatography and mass spectrometry. Anal. Chem..

[42] Derks T.G.J., Boer T.S., van Assen A., Bos T., Ruiter J., Waterham H.R., Niezen-Koning K.E., Wanders R.J.A., Rondeel J.M.M., Loeber J.G. (2008). Neonatal screening for medium-chain acyl-CoA dehydrogenase (MCAD) deficiency in The Netherlands: the importance of enzyme analysis to ascertain true MCAD deficiency. J. Inherit. Metab. Dis..

[43] Muskiet F.A., van Doormaal J.J., Martini I.A., Wolthers B.G., van der Slik W. (1983). Capillary gas chromatographic profiling of total long-chain fatty acids cholesterol in biological materials. J. Chromatogr..

[44] Lee W.N., Byerley L.O., Bergner E.A., Edmond J. (1991). Mass isotopomer analysis: Theoretical and practical considerations. Biol. Mass Spectrom..

[45] Dalla Man C., Caumo A., Basu R., Rizza R., Toffolo G., Cobelli C. (2004). Minimal model estimation of glucose absorption and insulin sensitivity from oral test: validation with a tracer method. Am. J. Physiol. Endocrinol. Metab..

[46] Soetaert K., Petzoldt T. (2010). Inverse modelling, sensitivity and Monte Carlo analysis in R using package FME. J. Stat. Software.

[47] Soetaert K., Petzoldt T., Setzer R.W. (2010). Solving differential equations in R: package deSolve. J. Stat. Software.

[48] An R. (2020).

[49] Fehlbaum S., Prudence K., Kieboom J., Heerikhuisen M., van den Broek T., Schuren F.H.J., Steinert R.E., Raederstorff D. (2018). In Vitro Fermentation of Selected Prebiotics and Their Effects on the Composition and Activity of the Adult Gut Microbiota. Int. J. Mol. Sci..

[50] Bach Knudsen K.E., Hessov I. (1995). Recovery of inulin from Jerusalem artichoke (Helianthus tuberosus L.) in the small intestine of man. Br. J. Nutr..

[51] Molis C., Flourié B., Ouarne F., Gailing M.F., Lartigue S., Guibert A., Bornet F., Galmiche J.P. (1996). Digestion, excretion, and energy value of fructooligosaccharides in healthy humans. Am. J. Clin. Nutr..

[52] Zoetendal E.G., Raes J., van den Bogert B., Arumugam M., Booijink C.C.G.M., Troost F.J., Bork P., Wels M., de Vos W.M., Kleerebezem M. (2012). The human small intestinal microbiota is driven by rapid uptake and conversion of simple carbohydrates. ISME J..

[53] Cummings J.H., Pomare E.W., Branch W.J., Naylor C.P., Macfarlane G.T. (1987). Short chain fatty acids in human large intestine, portal, hepatic and venous blood. Gut.

[54] Piche T., des Varannes S.B., Sacher-Huvelin S., Holst J.J., Cuber J.C., Galmiche J.P. (2003). Colonic fermentation influences lower esophageal sphincter function in gastroesophageal reflux disease. Gastroenterology.

[55] Hong K.B., Kim J.H., Kwon H.K., Han S.H., Park Y., Suh H.J. (2016). Evaluation of Prebiotic Effects of High-Purity Galactooligosaccharides *in vitro* and *in vivo*. Food Technol. Biotechnol..

[56] Savignac H.M., Corona G., Mills H., Chen L., Spencer J.P.E., Tzortzis G., Burnet P.W.J. (2013). Prebiotic feeding elevates central brain derived neurotrophic factor, N-methyl-D-aspartate receptor subunits and D-serine. Neurochem. Int..

[57] Koziolek M., Grimm M., Becker D., Iordanov V., Zou H., Shimizu J., Wanke C., Garbacz G., Weitschies W. (2015). Investigation of pH and temperature profiles in the GI tract of fasted human subjects using the Intellicap® system. J. Pharmaceut. Sci..

[58] Helander H.F., Fändriks L. (2014). Surface area of the digestive tract - revisited. Scand. J. Gastroenterol..

[59] El Aidy S., van den Bogert B., Kleerebezem M. (2015). The small intestine microbiota, nutritional modulation and relevance for health. Curr. Opin. Biotechnol..

[60] Booijink C.C.G.M., El-Aidy S., Rajilić-Stojanović M., Heilig H.G.H.J., Troost F.J., Smidt H., Kleerebezem M., De Vos W.M., Zoetendal E.G. (2010). High temporal and inter-individual variation detected in the human ileal microbiota. Environ. Microbiol..

[61] Seekatz A.M., Schnizlein M.K., Koenigsknecht M.J., Baker J.R., Hasler W.L., Bleske B.E., Young V.B., Sun D. (2019). Spatial and Temporal Analysis of the Stomach and Small-Intestinal Microbiota in Fasted Healthy Humans. mSphere.

[62] van Trijp M.P.H., Rösch C., An R., Keshtkar S., Logtenberg M.J., Hermes G.D.A., Zoetendal E.G., Schols H.A., Hooiveld G.J.E.J. (2020). Fermentation Kinetics of Selected Dietary Fibers by Human Small Intestinal Microbiota Depend on the Type of Fiber and Subject. Mol. Nutr. Food Res..

[63] Bouhnik Y., Neut C., Raskine L., Michel C., Riottot M., Andrieux C., Guillemot F., Dyard F., Flourié B. (2004). Prospective, randomized, parallel-group trial to evaluate the effects of lactulose and polyethylene glycol-4000 on colonic flora in chronic idiopathic constipation. Aliment. Pharmacol. Ther..

[64] Mangin I., Bouhnik Y., Suau A., Rochet V., Raskine L., Crenn P., Dyard F., Rambaud J.-C., Doré J. (2002). Molecular Analysis of Intestinal Microbiota Composition to Evaluate the Effect of PEG and Lactulose Laxatives in Humans. Microb. Ecol. Health Dis..

[65] Kerr B.J., Weber T.E., Ziemer C.J. (2015). Dietary marker effects on fecal microbial ecology, fecal VFA, nutrient digestibility coefficients, and growth performance in finishing pigs. J. Anim. Sci..

[66] Chen H., Zhao R., Wang B., Cai C., Zheng L., Wang H., Wang M., Ouyang H., Zhou X., Chai Z. (2017). The effects of orally administered Ag, TiO2 and SiO2 nanoparticles on gut microbiota composition and colitis induction in mice. NanoImpact.

[67] Pinget G., Tan J., Janac B., Kaakoush N.O., Angelatos A.S., O'Sullivan J., Koay Y.C., Sierro F., Davis J., Divakarla S.K. (2019). Corrigendum: Impact of the Food Additive Titanium Dioxide (E171) on Gut Microbiota-Host Interaction. Front. Nutr..

[68] Chen Z., Han S., Zhou D., Zhou S., Jia G. (2019). Effects of oral exposure to titanium dioxide nanoparticles on gut microbiota and gut-associated metabolism *in vivo*. Nanoscale.

[69] Dudefoi W., Moniz K., Allen-Vercoe E., Ropers M.H., Walker V.K. (2017). Impact of food grade and nano-TiO(2) particles on a human intestinal community. Food Chem. Toxicol..

[70] Radziwill-Bienkowska J.M., Talbot P., Kamphuis J.B.J., Robert V., Cartier C., Fourquaux I., Lentzen E., Audinot J.N., Jamme F., Réfrégiers M. (2018). Toxicity of Food-Grade TiO(2) to Commensal Intestinal and Transient Food-Borne Bacteria: New Insights Using Nano-SIMS and Synchrotron UV Fluorescence Imaging. Front. Microbiol..

[71] van den Bogert B. (2013).

[72] Roostalu J., Jõers A., Luidalepp H., Kaldalu N., Tenson T. (2008). Cell division in Escherichia colicultures monitored at single cell resolution. BMC Microbiol..

[73] Alsharafani M., Schnell S., Ratering S., Krawinkel M. (2016).

[74] Weaver J.C., Williams G.B., Klibanov A., Demain A.L. (1988). Gel microdroplets: rapid detection and enumeration of individual microorganisms by their metabolic activity. Nat. Biotechnol..

[75] Takeshita T., Kageyama S., Furuta M., Tsuboi H., Takeuchi K., Shibata Y., Shimazaki Y., Akifusa S., Ninomiya T., Kiyohara Y., Yamashita Y. (2016). Bacterial diversity in saliva and oral health-related conditions: the Hisayama Study. Sci. Rep..

[76] Nardone G., Compare D. (2015). The human gastric microbiota: Is it time to rethink the pathogenesis of stomach diseases?. United European Gastroenterol. J..

[77] Ruppin H., Bar-Meir S., Soergel K.H., Wood C.M., Schmitt M.G. (1980). Absorption of Short-Chain Fatty Acids by the Colon. Gastroenterology.

[78] Schmitt M.G., Soergel K.H., Wood C.M., Steff J.J. (1977). Absorption of short-chain fatty acids from the human ileum. Am. J. Dig. Dis..

[79] Weinman E.O., Strisower E.H., Chaikoff I.L. (1957). Conversion of Fatty Acids to Carbohydrate: Application of Isotopes to this Problem and Role of the Krebs Cycle as a Synthetic Pathway. Physiol. Rev..

[80] Bose S., Ramesh V., Locasale J.W. (2019). Acetate Metabolism in Physiology, Cancer, and Beyond. Trends Cell Biol..

[81] Astbury S.M., Corfe B.M. (2012). Uptake and metabolism of the short-chain fatty acid butyrate, a critical review of the literature. Curr. Drug Metabol..

[82] Bergman E.N. (1990). Energy contributions of volatile fatty acids from the gastrointestinal tract in various species. Physiol. Rev..

[83] De Vadder F., Kovatcheva-Datchary P., Goncalves D., Vinera J., Zitoun C., Duchampt A., Bäckhed F., Mithieux G. (2014). Microbiota-Generated Metabolites Promote Metabolic Benefits via Gut-Brain Neural Circuits. Cell.

[84] McBURNEY M.I., THOMPSON L.U. (1989). In Vitro Fermentabilities of Purified Fiber Supplements. J. Food Sci..

[85] McBurney M.I., Thompson L.U., Cuff D.J., Jenkins D.J. (1988). Comparison of ileal effluents, dietary fibers, and whole foods in predicting the physiological importance of colonic fermentation. Am. J. Gastroenterol..

[86] Cummings J.H., Macfarlane G.T. (1997). Colonic microflora: Nutrition and health. Nutrition.

