# Supplementary material for: Intraintestinal fermentation of fructo- and galacto-oligosaccharides and the fate of short-chain fatty acids in humans
Source: iScience. 2024 Feb 10;27(3):109208. doi: 10.1016/j.isci.2024.109208 (PMC10901090; doi:10.1016/j.isci.2024.109208)
Supplement: Document S1. Figures S1–S16 and Table S2 [file mmc1.pdf]

iScience, Volume *27*

Supplemental information

Intraintestinal fermentation

# of fructo- and galacto-oligosaccharides

and the fate of short-chain fatty acids in humans

Mara P.H. van Trijp, Melany Rios-Morales, Ben Witteman, Fentaw Abegaz, Albert Gerding, Ran An, Martijn Koehorst, Bernard Evers, Katja C.V. van Dongen, Erwin G. Zoetendal, Henk Schols, Lydia A. Afman, Dirk-Jan Reijngoud, Barbara M. Bakker, and Guido J. Hooiveld

Supplemental Information

Study logistics and drop-outs.

In study 1 during the first test session, two subjects were excluded due to failure of catheter placement in the ileum/colon (the catheter coiled up in the stomach). This resulted in adaptations in the catheter progression protocol, namely the balloon was kept inflated continuously and the insertion speed was reduced to a maximum (10 cm/hour). After these adaptations, the progression of the catheter occurred as expected, but one subject had to stop due to vomiting. Thus, from the five subjects, only two subjects fully completed study 1. In study 2, 10 subjects were included in the study and randomly allocated to the NDC (n=5) or placebo (n=5) group. One subject dropped out during the supplementation period due to the emergence of AEs, namely diarrhea. Three subjects were excluded due to failure of post-pyloric catheter placement. The remaining six subjects completed study 2, and compliance was 101±4.9% for the different intervention products, based on percentages of product packages returned. The percentage is higher than 100, because one subject incidentally also consumed the extra supplement that was provided. The total radiation effective dose that subjects were exposed to while verifying the catheter positioning using fluoroscopy was a calculated mean of 0.06 (range 0.005-0.22) mSv, which can be considered very low. The dose varied upon the exposure time to fluoroscopy, mainly depending on the time the placement procedure took.

Evaluation of (dis)comfort.

The study procedures caused expected discomfort and AEs. Only in study 2, the participants were asked to fill in a questionnaire with visual analog scales about the study procedures, labeled with ‘no pain/discomfort’ (0 mm end) and ‘a lot of pain/discomfort’ (100 mm end). Nine subjects that had the catheter inserted graded throat pain with 33±22 mm, nasal pain with 38±23 mm, and nausea with 28±37 mm. Catheter placement was graded with 45±24 mm, catheter progression with 29±24 mm (n=7), and catheter removal with 51±33 mm. 67% of the participants indicated that they would undergo the same procedure again for clinical research, but only 33% of the participants indicated that they would undergo this procedure twice within two months.

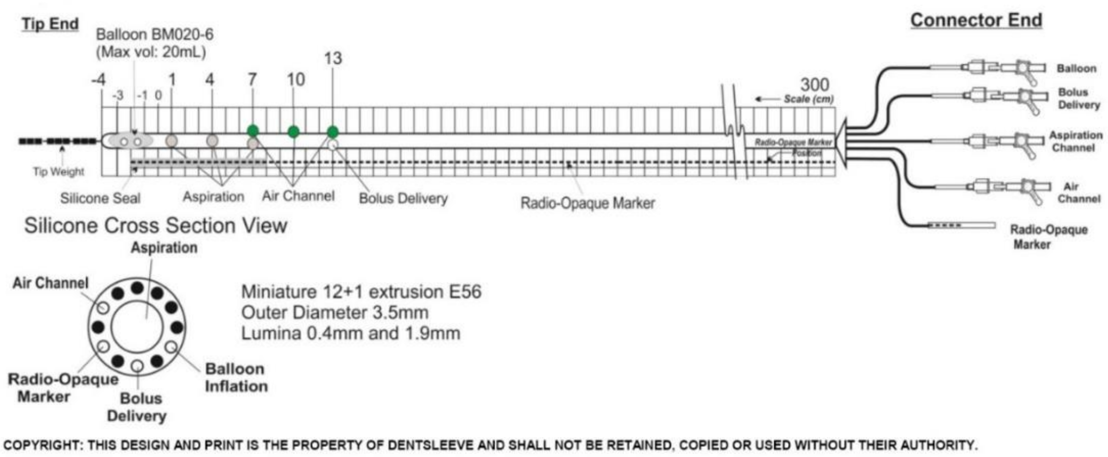

Figure S1. Design of the naso-intestinal catheter, related to STAR methods. *In the cross-section view, the different lumina are shown. The radio-opaque marker is used for visualization by fluoroscopy.*

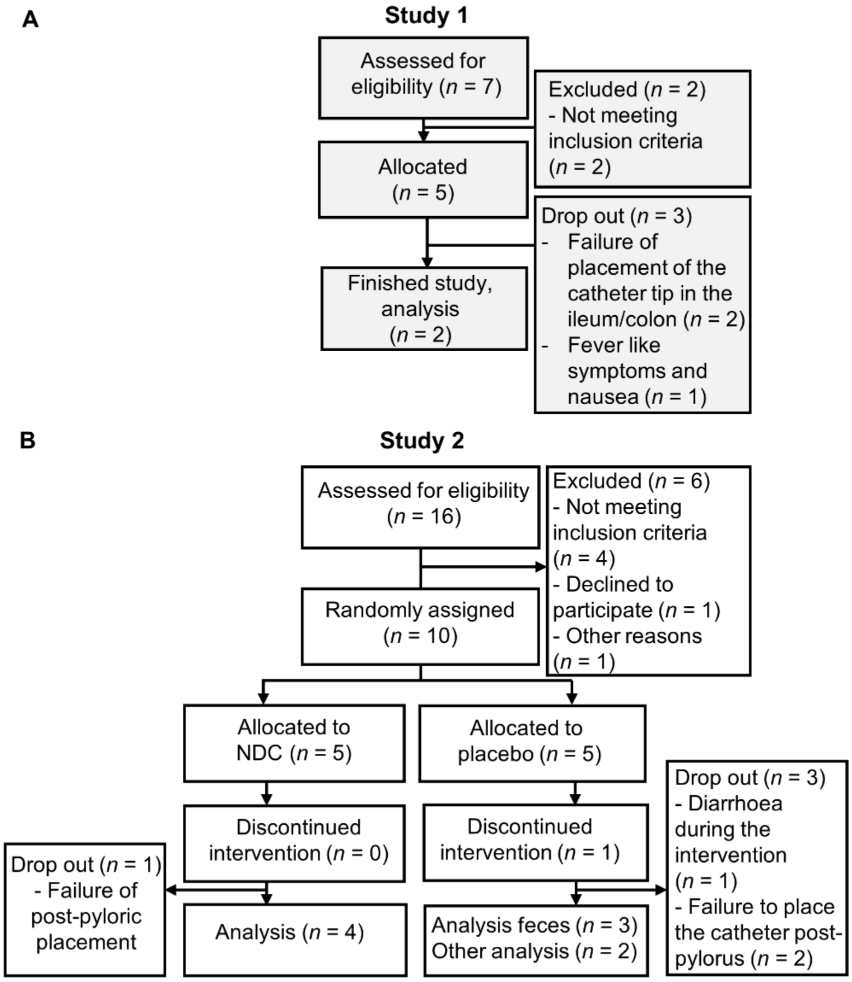

Figure S2. Flow chart of study 1 and study 2, related to Table 1.

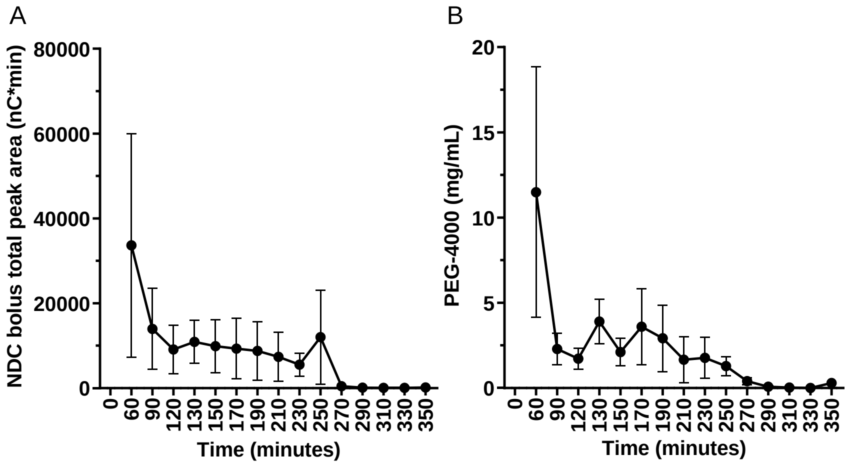

Figure S3. The presence of NDC constituents (A) or the non-absorbable marker PEG-4000 (B) in the ileum or proximal colon of healthy male subjects over time, related to Figure 3. *Data is shown as mean ± SD, n=7 subjects in study 1 and study 2. The data of the transverse colon, is not included in this figure.*

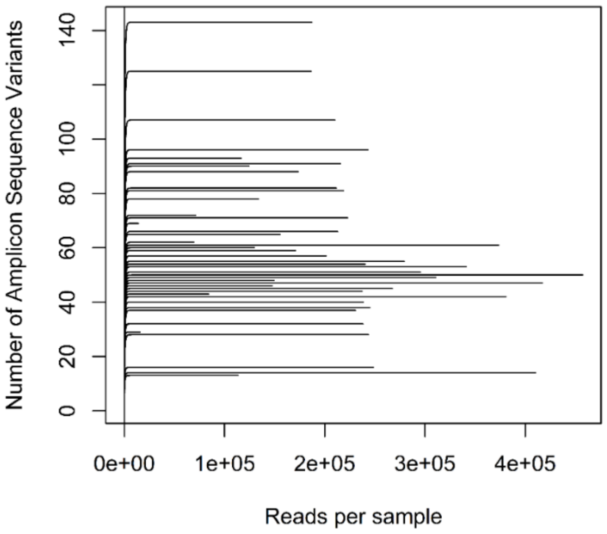

Figure S4. The rarefaction curves of all intestinal and fecal samples, related to Figure 2*. When the curve reaches a plateau, this means the sequencing depth (reads per sample) was high enough to measure all amplicon sequence variants (bacteria) present in the sample. Control samples (water, TiO2 and MOCKs) are excluded from this figure.*

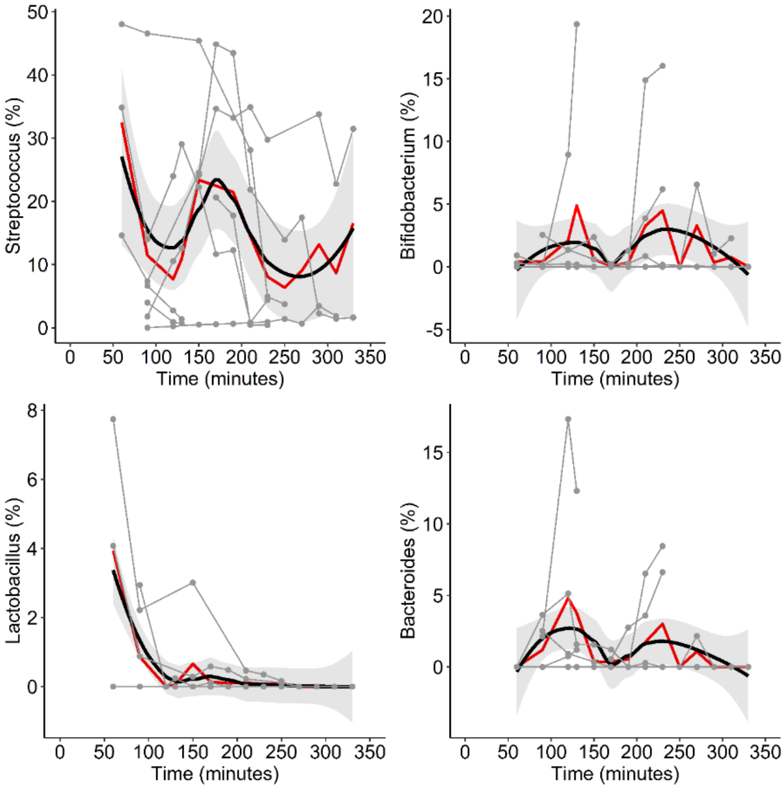

Figure S5. The relative abundances of the selected bacteria Streptococcus, Bifidobacterium, Lactobacillus, and Bacteroides over time inside the ileum or colon of healthy male subjects, related to Figure 5. *The grey dots and lines represent the individual patterns. The red line represents the mean relative abundance, and the black line represents the locally estimated scatterplot smoothing curve. Data is presented from n = 8 subjects in study 1 and study 2.*

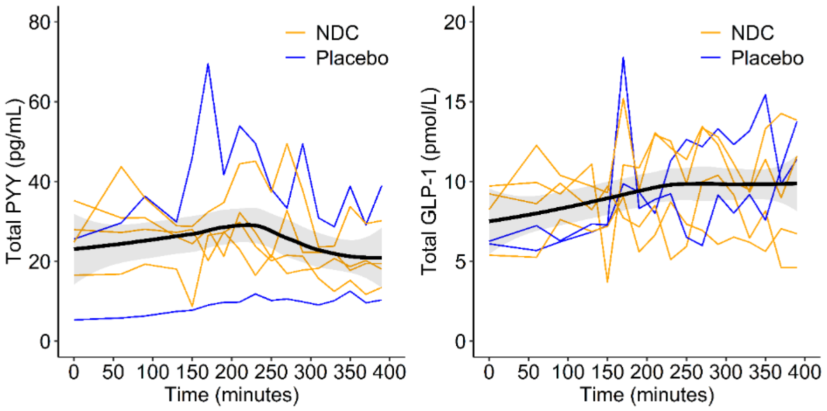

Figure S6. Concentrations of total PYY and total GLP-1 in plasma of healthy male subjects before and after consumption of the NDC bolus with 10 grams FOS and GOS, related to Figure 7. *The lines represent the individual patterns. The black line represents the locally estimated scatterplot smoothing curve (n=6 subjects in study 2). PYY, peptide YY; GLP-1, glucagon-like peptide 1; NDC, non-digestible carbohydrates.*

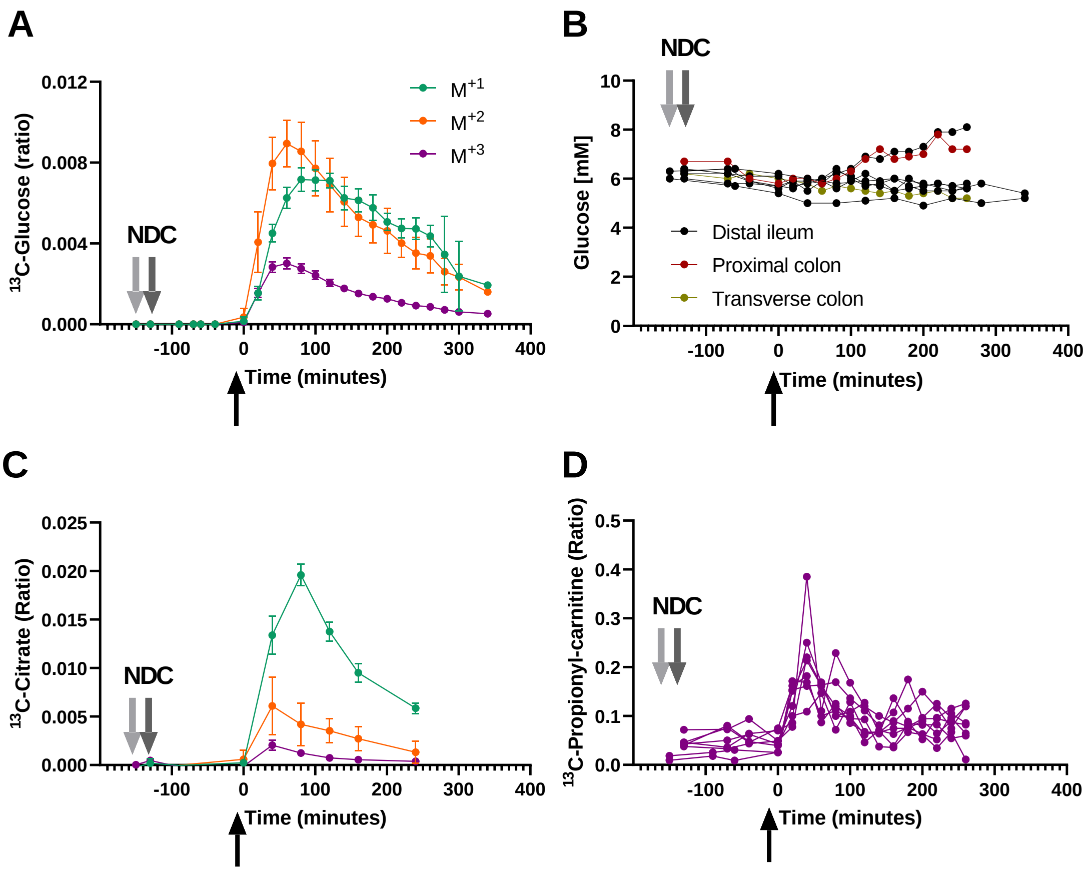

Figure S7. The glucose and citrate enrichment ratios per labeled pattern (M^0-M^{+5}, M^{+6} not detected) from SCFA delivered in the intestine, and the total plasma glucose concentration over time, related to Figure 7. *(A) Glucose enrichments per label pattern detected and (B) blood glucose concentrations. (C) Citrate enrichments per label pattern detected. (D) Propionyl-carnitine enrichments per label pattern detected. The black arrow indicates the start of luminal isotope infusion (10 ml containing 10 mmoles [1-^{13}C]-acetate, 4 mmoles [1,2,3-$^{13}C_3$]-propionate and 1 mmol [1,2,3,4-$^{13}C_4$]-butyrate) through the catheter. This is considered to be time 0 for all subjects to match both studies. The grey arrows indicate the drinking of the non-digestible carbohydrates (NDC) bolus in both studies (-150 min for study 1, light grey arrow, and -120 min for study 2, dark grey arrow). Data is represented as mean (A, C) and n=8 individual subjects (B,D).*

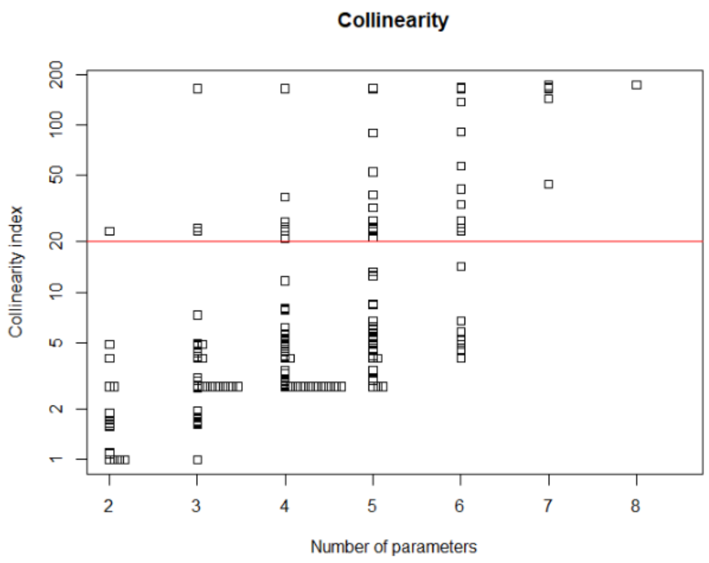

Figure S8. Multivariate parameter identifiability approach for the eight parameters included in the model, using the average data of all subjects, related to Figure 9*. A collinearity index of less than 20 is indicated by the red cut-off line. Each square represents a different combination of parameters.*

Parameter (min^{-1})	**Estimate**	**Std. Error**	**p-value**
k_{La}	0.0015	0.0004	2.11e-4 ***
k_{1p}	0.0043	0.0002	< 2e-16 ***
k_{1b}	0.0261	0.0021	< 2e-16 ***
k_2	0.0104	0.0006	< 2e-16 ***

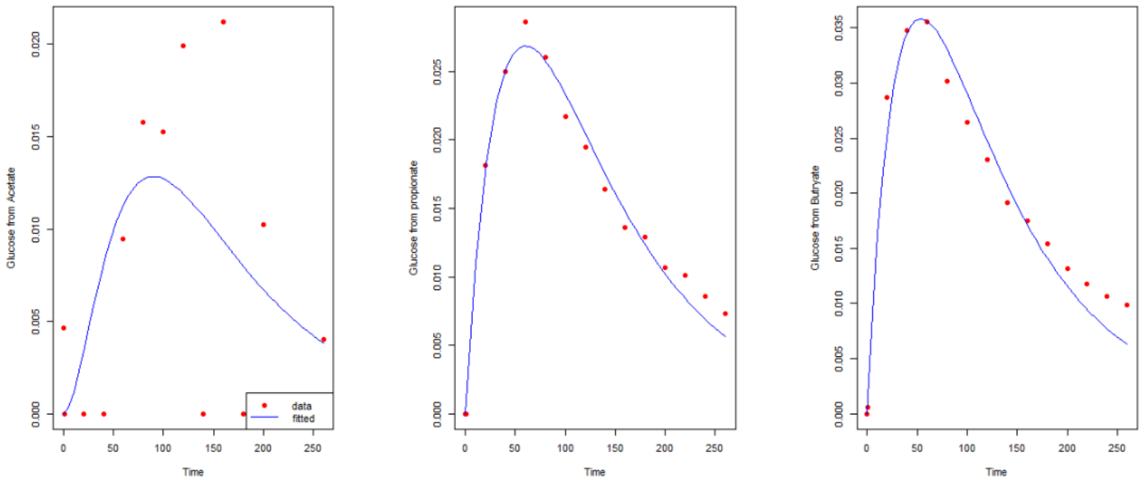

Figure S9. Model fitting for subject 1, related to Figure 9. *Experimentally measured values (in red) and fitted values (in blue) for the incorporation of acetate, propionate, and butyrate into blood glucose. *p<0.05. **p<0.01. ***p<0.001.*

Parameter (min^{-1})	**Estimate**	**Std. Error**	**p-value**
k_{La}	0.0021	0.0002	< 2e-16 ***
k_{1p}	0.0037	0.0002	< 2e-16 ***
k_{1b}	0.0161	0.0008	< 2e-16 ***
k_{2}	0.0133	0.0005	< 2e-16 ***

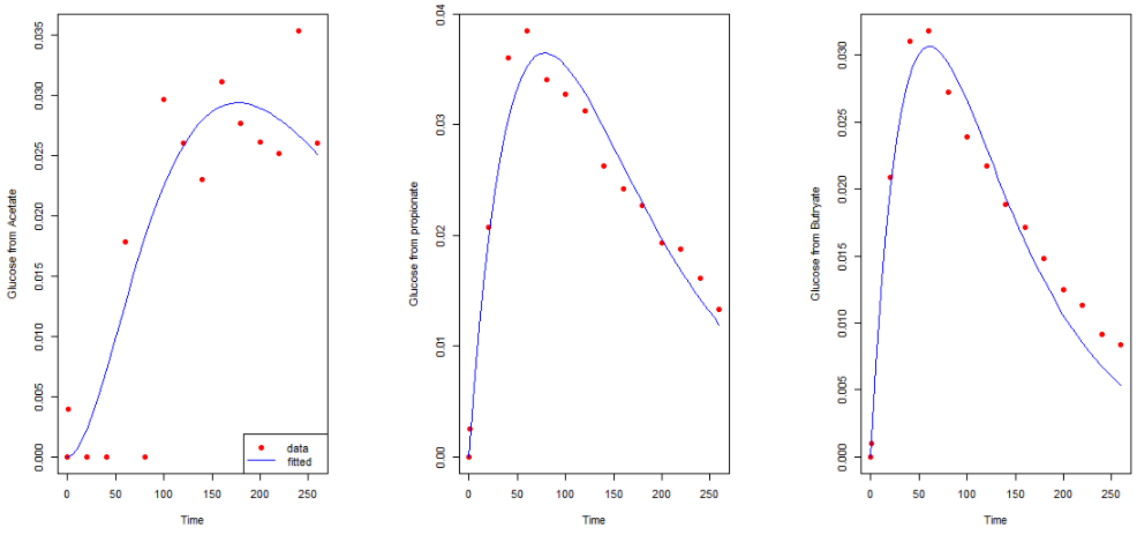

Figure S10. Model fitting for subject 2, related to Figure 9. *Experimentally measured values (in red) and fitted values (in blue) for the incorporation of acetate, propionate, and butyrate into blood glucose. *p<0.05. **p<0.01. ***p<0.001.*

Parameter (min^{-1})	**Estimate**	**Std. Error**	**p-value**
k_{La}	0.0027	0.0003	< 2e-16 ***
k_{1p}	0.0039	0.0001	< 2e-16 ***
k_{1b}	0.0251	0.0013	< 2e-16 ***
k_2	0.0070	0.0002	< 2e-16 ***

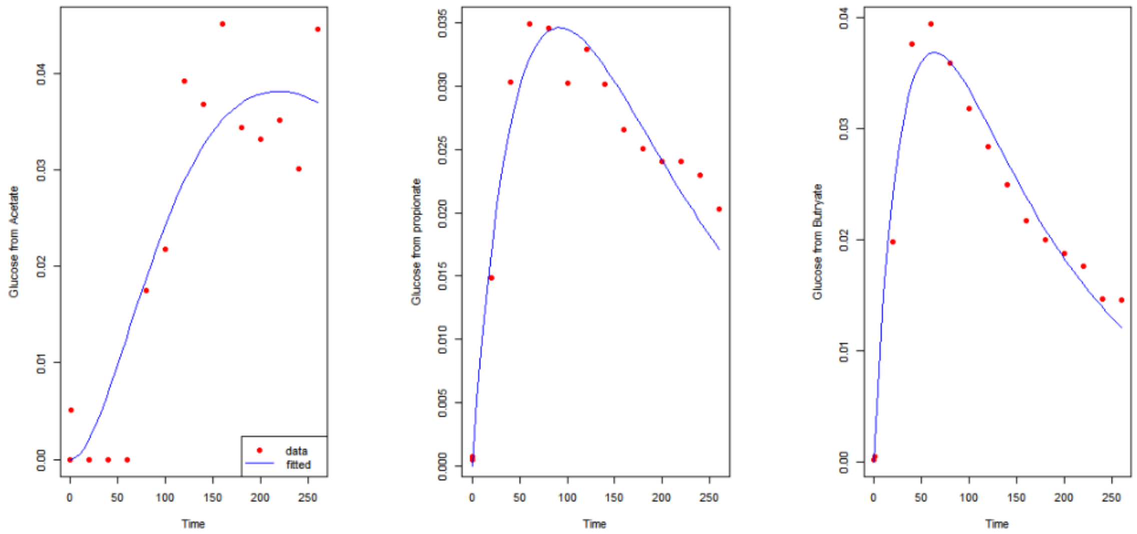

Figure S11. Model fitting for subject 3, related to Figure 9. *Experimentally measured values (in red) and fitted values (in blue) for the incorporation of acetate, propionate, and butyrate into blood glucose. *p<0.05. **p<0.01. ***p<0.001.*

Parameter (min^{-1})	Estimate	Std. Error	p-value
k_{La}	0.0025	0.0005	3.36e-06 ***
k_{1p}	0.0031	0.0002	< 2e-16 ***
k_{1b}	0.0236	0.0017	< 2e-16 ***
k_2	0.0072	0.0003	< 2e-16 ***

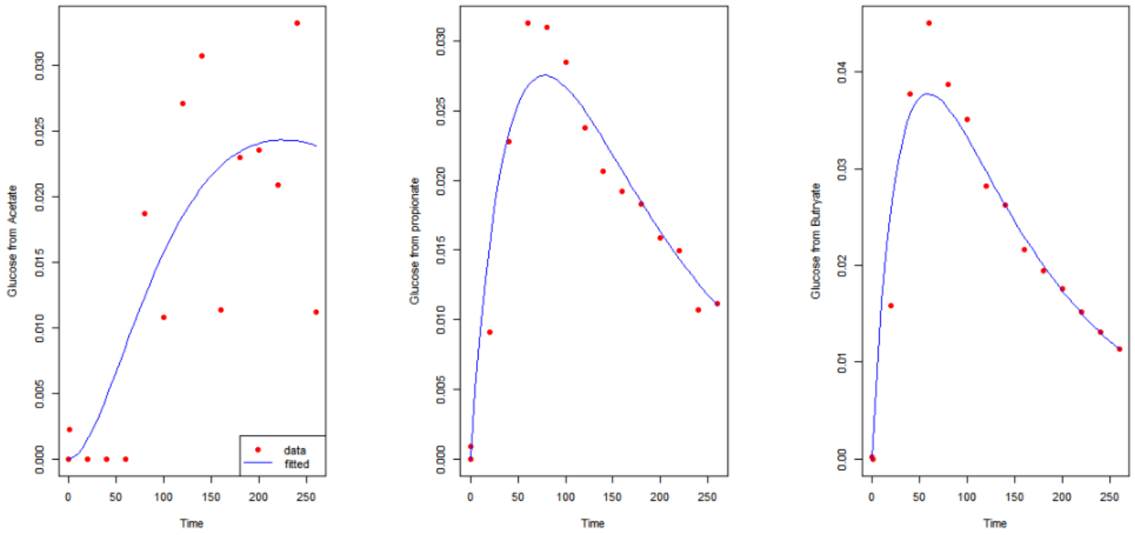

Figure S12. Model fitting for subject 4, related to Figure 9. *Experimentally measured values (in red) and fitted values (in blue) for the incorporation of acetate, propionate, and butyrate into blood glucose. *p<0.05. **p<0.01. ***p<0.001.*

Parameter (min^{-1})	Estimate	Std. Error	p-value
k_{La}	0.0005	0.0001	1.99e-13 ***
k_{1p}	0.0027	0.0002	< 2e-16 ***
k_{1b}	0.0105	0.0007	< 2e-16 ***
k_2	0.0117	0.0021	< 2e-16 ***

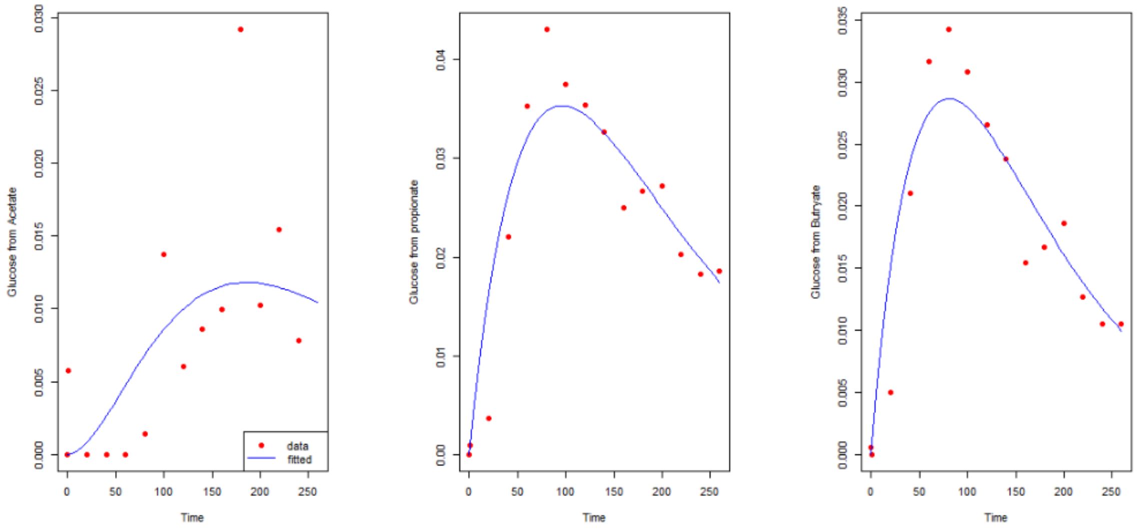

Figure S13. Model fitting for subject 5, related to Figure 9. *Experimentally measured values (in red) and fitted values (in blue) for the incorporation of acetate, propionate, and butyrate into blood glucose. *p<0.05. **p<0.01. ***p<0.001.*

Parameter (min^{-1})	Estimate	Std. Error	p-value
k_{La}	0.0026	0.0002	< 2e-16 ***
k_{1p}	0.0042	0.0002	< 2e-16 ***
k_{1b}	0.0238	0.0014	< 2e-16 ***
k_2	0.0097	0.0004	< 2e-16 ***

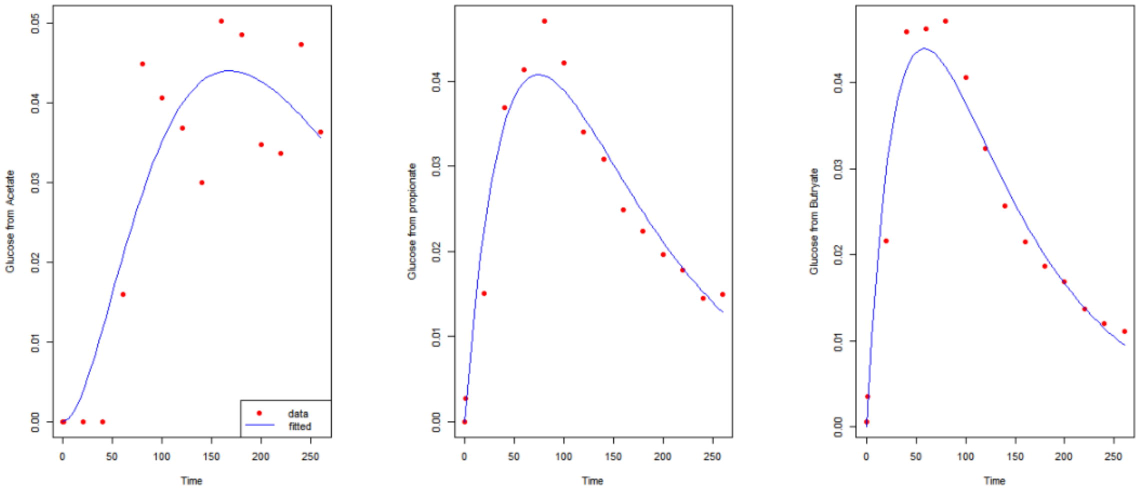

Figure S14. Model fitting for subject 6, related to Figure 9. *Experimentally measured values (in red) and fitted values (in blue) for the incorporation of acetate, propionate, and butyrate into blood glucose. *p<0.05. **p<0.01. ***p<0.001.*

Parameter ***(min^{-1})***	**Estimate**	**Std. Error**	**p-value**
k_{La}	0.0023	0.0025	3.56e-01
k_{1p}	0.0031	0.0001	< 2e-16 ***
k_{1b}	0.0146	0.0017	7.66e-15 ***
k_2	0.0050	0.0003	< 2e-16 ***

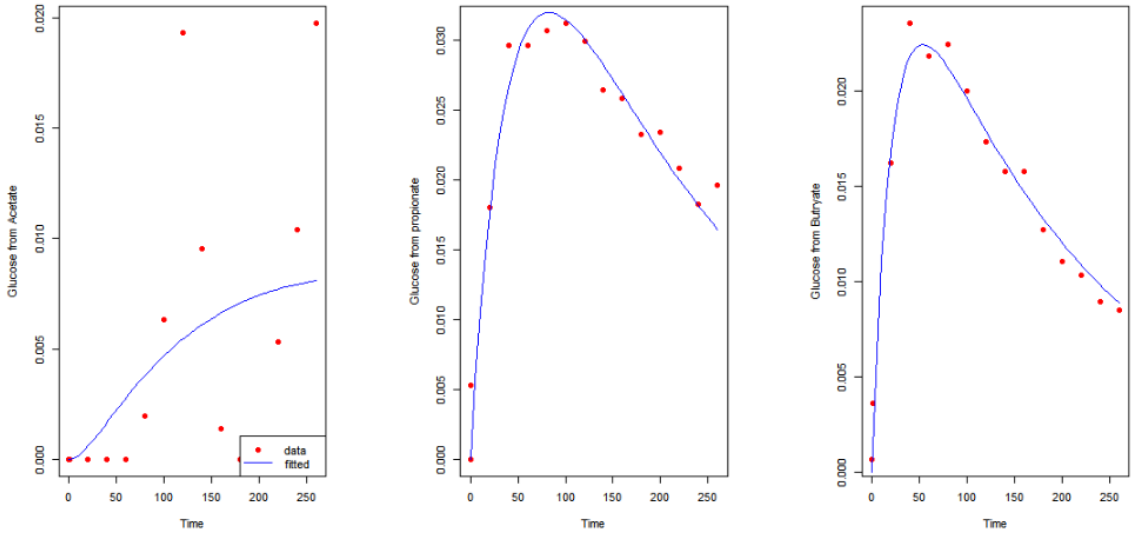

Figure S15. Model fitting for subject 7, related to Figure 9. *Experimentally measured values (in red) and fitted values (in blue) for the incorporation of acetate, propionate, and butyrate into blood. *p<0.05. **p<0.01. ***p<0.001.*

Parameter (min^{-1})	Estimate	Std. Error	p-value
k_{La}	0.0027	0.0002	< 2e-16 ***
k_{1p}	0.0046	0.0001	< 2e-16 ***
k_{1b}	0.0186	0.0015	< 2e-16 ***
k_2	0.0067	0.0002	< 2e-16 ***

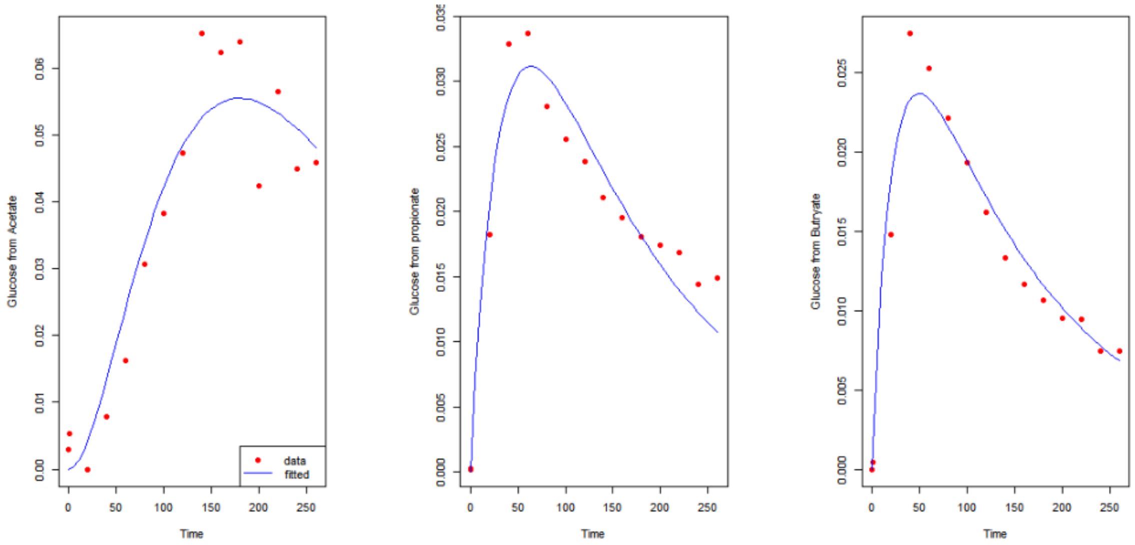

Figure S16. Model fitting for subject 8, related to Figure 9. *Experimentally measured values (in red), and fitted values (in blue) for the incorporation of acetate, propionate, and butyrate into blood. *p<0.05. **p<0.01. ***p<0.001.*

Table S2. The differential fecal bacteria (P-values<0.05) and short-chain fatty acids in feces of healthy male subjects after 7-days supplementation, related to Figure 2. *The NDC group received 15 g/day FOS:GOS for 7 days, and the placebo group received isocaloric maltodextrin.*

	**NDC group (*n* = 4)**	**Placebo group (*n* = 3)**		
**Bacteria on genus level**	**Relative abundance (%)**	**Relative abundance (%)**	**P-value**	**FDR P-value**
g__Holdemanella	0 ± 0	1.98 ± 3.66	0.0319	N.S.
g__Coprococcus_3	0.112 ± 0.135	0.337 ± 0.332	0.0497	N.S.
g__Ruminococcaceae_NK4A214_group	0.0841 ± 0.112	0.483 ± 0.591	0.0497	N.S.
**Short-chain fatty acid**	**Concentration (mM)**	**Concentration (mM)**	**P-value**	
Acetate	120.6 ± 78.3	96.0 ± 67.8	0.289	-
Propionate	36.8 ± 20.2	30.9 ± 18.0	0.289	-
Butyrate	29.6 ± 21.2	23.8 ± 17.3	0.157	-
